# Unlocking bacterial potential to reduce farmland N_2_O emissions

**DOI:** 10.1038/s41586-024-07464-3

**Published:** 2024-05-29

**Authors:** Elisabeth G. Hiis, Silas H. W. Vick, Lars Molstad, Kristine Røsdal, Kjell Rune Jonassen, Wilfried Winiwarter, Lars R. Bakken

**Affiliations:** 1 Faculty of Chemistry, Biotechnology and Food Science, Norwegian University of Life Sciences, Ås, Norway; 2 Veas WWTP, Slemmestad, Norway; 3 International Institute for Applied Systems Analysis, Laxenburg, Austria; 4 Institute of Environmental Engineering, University of Zielona Góra, Zielona Góra, Poland

**Keywords:** Climate sciences, Biogeochemistry

## Abstract

Farmed soils contribute substantially to global warming by emitting N_2_O (ref. ^1^), and mitigation has proved difficult^2^. Several microbial nitrogen transformations produce N_2_O, but the only biological sink for N_2_O is the enzyme NosZ, catalysing the reduction of N_2_O to N_2_ (ref. ^3^). Although strengthening the NosZ activity in soils would reduce N_2_O emissions, such bioengineering of the soil microbiota is considered challenging^4,5^. However, we have developed a technology to achieve this, using organic waste as a substrate and vector for N_2_O-respiring bacteria selected for their capacity to thrive in soil^6–8^. Here we have analysed the biokinetics of N_2_O reduction by our most promising N_2_O-respiring bacterium, *Cloacibacterium* sp. CB-01, its survival in soil and its effect on N_2_O emissions in field experiments. Fertilization with waste from biogas production, in which CB-01 had grown aerobically to about 6 × 10^9^ cells per millilitre, reduced N_2_O emissions by 50–95%, depending on soil type. The strong and long-lasting effect of CB-01 is ascribed to its tenacity in soil, rather than its biokinetic parameters, which were inferior to those of other strains of N_2_O-respiring bacteria. Scaling our data up to the European level, we find that national anthropogenic N_2_O emissions could be reduced by 5–20%, and more if including other organic wastes. This opens an avenue for cost-effective reduction of N_2_O emissions for which other mitigation options are lacking at present.

## Abstract (web summary)

A study presents a method to mitigate emissions of nitrous oxide from farmland using bacteria to consume nitrous oxide in soil with organic waste as a substrate and vector.

## Main

Until the mid-twentieth century, crop production was severely limited by nitrogen, requiring farmers to recycle this element in a reactive form within their agroecosystems. This constraint is reflected in the agricultural treatise by Marcus Porcius Cato (234–143 bc) *De Agri Cultura*, which recommends to “save carefully goat, sheep, cattle, and all other dung”^9^. The invention of the Haber–Bosch process in 1908 eliminated the nitrogen constraint by producing ammonium from atmospheric nitrogen. The Haber–Bosch process was a breakthrough, saving the world from starvation^10^, but has also become a problem because it allowed farmers to use nitrogen in excess, with marginal economic penalties for losing nitrogen to the environment. As a result, most agroecosystems have become nitrogen-enriched and leaky, releasing ammonia to the atmosphere and nitrate to the groundwater and surface water, at scales that induce eutrophication and threaten the quality and resilience of both terrestrial and aquatic ecosystems worldwide^2,11–13^. The global scale of the problem becomes apparent when considering that the flux of reactive nitrogen into the biosphere has practically doubled since the industrial revolution, primarily owing to nitrogen produced through the Haber–Bosch process^14^.

Nitrogen fertilization causes emissions of the greenhouse gas N_2_O, both from agricultural soils themselves (direct emissions) and from the natural environments owing to the input of reactive nitrogen lost from the farms (indirect emissions). These farming-induced emissions account for substantial shares of the escalating concentration of N_2_O in the atmosphere since the industrial revolution^1,15,16^. A comprehensive analysis of global N_2_O emissions for 2007–2016^17^ estimated that total direct and indirect emissions were 2.3–5.2 and 0.6–2.1 Tg N_2_O-N yr^−1^, respectively, in total accounting for >50% of the total anthropogenic N_2_O emissions (4.1–10.3 Tg N_2_O-N yr^−1^).

## Mitigation

Reducing the anthropogenic impacts on nitrogen cycling and N_2_O emissions has become a major environmental challenge for the twenty-first century owing to the severity of these issues. An obvious place to start is to improve the nitrogen-use efficiency of agroecosystems by reducing their losses of ammonia and nitrate^12^. This can be achieved by policy instruments to induce shifts in existing farming technologies and implementation of emerging ones^13,18–20^.

Although improving nitrogen-use efficiency can reduce emissions, deliberately manipulating the soil microorganisms holds even greater potential for achieving substantial reductions. N_2_O emitted from soils is produced by denitrifying bacteria, denitrifying fungi, ammonia-oxidizing archaea, ammonia-oxidizing bacteria^5^ and abiotic chemical reactions^21^. Whereas ammonia-oxidizing archaea, ammonia-oxidizing bacteria and denitrifying fungi are net sources of N_2_O because they lack the enzyme N_2_O reductase, denitrifying bacteria can be either sinks, sources or both: N_2_O is a free intermediate in their stepwise reduction of nitrate to molecular nitrogen, NO_3_^−^ to NO_2_^−^ to NO to N_2_O to N_2_, catalysed by enzymes encoded by the genes *nar* and *nap*; *nirS* and *nirK*; *cNor* and *qNor*; and *nosZ*, respectively^3^. The organisms use this pathway to sustain their respiratory metabolism under hypoxic and anoxic conditions. Denitrifying bacteria are extremely diverse regarding their catabolic potential, their regulation of denitrification^22,23^ and their denitrification gene sets: a substantial share of denitrifying bacteria in soils have truncated denitrification pathways, lacking one to three of the four genes coding for the complete pathway^23,24^. This has been taken to suggest that denitrification is essentially ‘modular’ (that is, that each step of the pathway is catalysed by a separate group of organisms, rather than by organisms carrying out all of the steps of the pathway)^25^. The truth is probably a bit of both^4,26^. Of note, an organism with a truncated denitrification pathway lacking *nirS* and *nirK* is not a denitrifying bacterium sensu stricto.

Being the only sink for N_2_O in soils, the enzyme N_2_O reductase (NosZ) has been the target for recent attempts to mitigate N_2_O emissions from soils. An intervention that strengthens this sink will lower the N_2_O/N_2_ product ratio of denitrification and hence reduce the propensity of the soil to emit N_2_O into the atmosphere^5,27^. This can be achieved by liming to increase the soil pH: the synthesis of functional NosZ is enhanced by pushing the soil pH towards the upper end of the normal pH range of farmed soils (pH 5–7)^28^. As a result, liming acidified soils will reduce their N_2_O emissions by 10–20%, albeit with a next-to-neutral climate effect owing to the CO_2_ emission induced by lime application^29,30^.

## N_2_O-respiring bacteria

Increasing the abundance of N_2_O-respiring bacteria (NRB; Box 1) could decrease the emission of N_2_O (ref. ^31^). NRB with a complete denitrification pathway can be net sinks of N_2_O if their denitrification regulatory networks secure earlier and/or stronger expression of NosZ than of the other denitrification enzymes^6,32^, or if their electron flow is channelled preferentially to NosZ (ref. ^33^). Their effect as N_2_O sinks is plausibly conditional, however, as regulation of their anaerobic respiratory pathway can be influenced by environmental conditions. By contrast, bacteria that are equipped with *nosZ*, but lack *nirS* and *nirK*, are more likely to be effective sinks for N_2_O (ref. ^34^). In the following, we will call them non-denitrifying NRB (NNRB) because they are unable to denitrify, sensu stricto (Box 1). NNRB are sinks for N_2_O in hypoxia and anoxia, unless equipped with enzymes catalysing nitrate ammonification (that is, reduction of NO_3_^−^ to NH_4_^+^ via NO_2_^−^). Such NNRB organisms catalysing nitrate ammonification have been found to produce significant amounts of N_2_O if provided with high nitrate concentrations^35^; or when using Fe^3+^ as electron acceptor, thus inducing abiotic N_2_O formation by chemical reaction of Fe^2+^ with NO_2_^−^ (ref. ^21^).

We know too little about the ecology and physiology of NNRB to selectively enhance their growth in situ^4^, but their potential as agents to reduce N_2_O emissions from soils is indisputable, as demonstrated by laboratory incubations of soils amended with NNRB grown ex situ^36^. Recently, it was suggested^6^ that such soil amendment can be carried out inexpensively on a large scale, by using waste from biogas reactors (digestates), destined for soils as organic fertilizers, both as a substrate and vector for NRB or NNRB. By anoxic enrichment culturing with N_2_O as the sole electron acceptor, these authors successfully enriched and isolated NRB with a strong preference for N_2_O, which could grow aerobically to high cell densities in digestates, and showed that amending soils with NRB-enriched digestates lowered the N_2_O/N_2_ product ratio of denitrification. The isolates obtained were not ideal, however, because they had genes for the entire denitrification pathway, and their catabolic capacities were streamlined for growth in digestate, not soil. In a follow-up study^7^, the authors designed a dual substrate enrichment strategy, switching between sterilized digestate and soil as substrates, to deliberately select for NRB and NNRB with a broader catabolic capacity and physiochemical tolerance. The enrichments became dominated by strains classified as *Cloacibacterium* (based on 16S rRNA gene amplicon sequencing), and the isolated strain *Cloacibacterium* sp. CB-01 was deemed promising: it carries the genes for reduction of NO and N_2_O but lacks the genes for reduction of NO_3_^–^ and NO_2_^−^, thus qualifying as an NNRB (Box 1). A subsequent meta-omics analysis of the enrichments and the genome of CB-01 suggested that surface attachment and utilization of complex polysaccharides contributed to its fitness in soil^8^.

Here we have evaluated the ability of CB-01 to reduce N_2_O emission from soil, when vectored by digestate. We examined several regulatory and enzyme kinetic traits to assess its inherent strength as an N_2_O sink. We then tested its capacity in ‘real life’ by conducting field experiments in which soils were fertilized with digestate in which CB-01 had been grown to a high cell density. Last, we assessed the potential of this technology for reducing N_2_O emissions across the European Union.

Box 1 NRB and NNRB as bacterial sinks for N_2_O Bacteria with a complete denitrification pathway sustain their anaerobic respiration by stepwise reduction of NO_3_^−^ to N_2_, catalysed by four reductases, producing NO_2_^−^, NO and N_2_O as free intermediates:
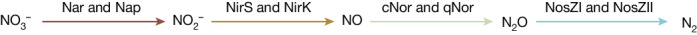

Many bacteria have a truncated pathway, lacking one to three of the reductase genes, with consequences for their role as sources or sinks for N_2_O.

**Terminology**

NRB: N_2_O-respiring bacteria. NRB equipped with *nirS* and *nirK* and *cNor* and *qNor* are either sinks or sources for N_2_O, depending on the regulatory network controlling their anaerobic respiration.

NNRB: non-denitrifying NRB. NNRB are NRB lacking the genes for denitrification sensu stricto (that is, *nirS* and *nirK*).

**N**_**2**_ **O reductase types**

There are two known versions of this copper enzyme: NosZI and NosZII.

Electrons are transferred to NosZII through a pathway other than NosZI, apparently generating more proton-motive force per electron^38,39^.

NosZII seems to have a higher affinity for N_2_O (refs. ^38,39^).

## The respiratory phenotype

The genome of CB-01 contains *nosZII* but lacks any genes coding for dissimilatory reduction of NO_3_^−^ and NO_2_^−^, predicting a phenotype able to respire N_2_O (but neither NO_3_^−^ nor NO_2_^−^), which was confirmed experimentally. In response to oxygen depletion, CB-01 reduced N_2_O to N_2_, but was unable to produce N_2_O from NO_2_^−^(ref. ^7^). The fact that it has *cNor*, coding for NO reductase, means that it could produce N_2_O from NO, but the NO kinetics indicates minor NO reductase activity^7^. This qualifies CB-01 as an NNRB (Box 1), and the laboratory incubation of soils fertilized with digestates containing CB-01 produced marginal amounts of N_2_O (ref. ^7^).

The capacity of a strain to reduce N_2_O emissions is commonly judged by a set of biokinetic parameters^31^, and we investigated these for CB-01, for comparison with other strains. In all experiments (unless otherwise stated), CB-01 was grown as batch cultures in GranuCult nutrient broth (Merck) containing meat peptone and meat extract, at pH 7.3 and 23 °C.

### Growth yield

**Extended Data Fig. 1:**
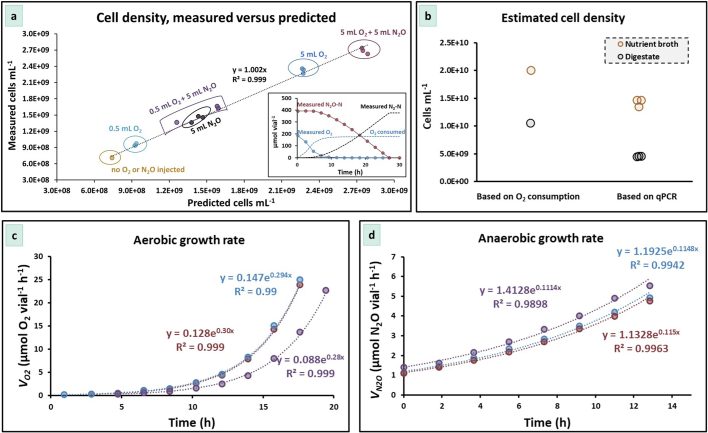
Growth yield and growth rates by aerobic and anaerobic respiration. **Panel a:** The growth yield of CB-01 by aerobic and anaerobic respiration assessed by batch cultivation in 50 mL nutrient broth (meat-peptone and meat extract) in 120 mL vials (crimp-sealed with butyl-rubber septa) with He-atmosphere, provided with N_2_O and O_2_. Vials were placed in the thermostatic water-bath (23 °C) of the robotized incubation system^54,55^. After temperature equilibration and subsequent release of overpressure due to N_2_O- and O_2_-injection, the vials were inoculated (3.5 × 10^10^ cells vial^−1^). Based on measured O_2_, N_2_O and N_2_ in the headspace, the cumulated reduction of O_2_ and N_2_O was estimated. The inserted panel shows an example of the gas kinetics in a single vial. When O_2_ and N_2_O had been depleted, the cell density was measured by OD_600_. The relationship between cell density and OD_600_ was determined in a separate experiment comparing OD_600_ with microscopic counts. A linear relationship was found for OD_600_ ≤ 0.5 (cell density = 3.34 × 10^9^ mL^−1^ OD^−1^). The cell dry weight, determined by weighing (cells washed three times in distilled water by dispersion and centrifugation, then dried at 105 °C), was 108 fg cell^−1^ ± s.e. = 7.5 (n = 9). The measured yield per mol of N_2_O and O_2_ was found by using the Generalized Reduced Gradient Solver in Excel (Microsoft Office 365, v2309) for the entire dataset. The panel shows the result for individual vials, as a plot of the predicted cell density (based on the yields given below) against measured cell density. The estimated yields were *Y*_*N2O*_ = 1.7 × 10^14^ cells mol^−1^ N_2_O and *Y*_*O2*_ = 4 × 10^14^ cells mol^−1^ O_2_. The yields per mol electrons are *Y*_*e-N2O*_ = 0.85 × 10^14^ mol^−1^ e^−^ to N_2_O and *Y*_*e-O2*_ = 1.0 × 10^14^ cells mol^−1^ e^−^ to O_2_. The yields in terms of dry weight g are ***Y***_***e-N2O***_ = **9.2** ± **0.6** **g** **mol**^**−1**^ **e**^**−**^ **to N**_**2**_**O and** ***Y***_***e-O2***_ = **11** ± **0.8** **g** **mol**^**−1**^ **e**^**−**^ **to O**_**2**_. In comparison, Bergaust et al. ^58^ found *Paracoccus denitrificans* to have *Y*_*e*_*-*_*O2*_ = 3.75 × 10^13^ cells mol^−1^ e^−^ = 11.2 g cell dry weight mol^−1^ e^−^ to O_2_ (cell dry weight = 298 fg), which is practically identical to *Y*_*e-O2*_ for CB-01. *Y*_*e-N2O*_ was 85% of Y_e-O2_ for CB-01, which is high compared to that measured for *P. denitrificans* (53%), and compared to the expectations ( ~ 60%) based on the charge separation per electron for aerobic and anaerobic respiration for NosZ clade I^37^. However, there is mounting evidence that the electron pathway to NosZ Clade II generates more charge separations than the pathway to NosZ Clade I, which is thermodynamically possible^31,38,96^. **Panel b:** The growth yield is plausibly declining as a culture reach stationary phase by depleting the C-sources. To assess the growth yields under these conditions, we quantified the cell densities by real-time quantitative PCR (qPCR), and compared this with the estimated cell densities based on the oxygen consumption and *Y*_*O2*_ = 4 × 10^14^ cells mol^−1^ O_2_. The panel shows this comparison for aerobic growth to high cell densities in nutrient broth and digestate, confirming lower *Y*_*O2*_ when the cultures approach stationary phase, more so in digestate than in nutrient broth. We find that *Y*_*O2*_ for growth in digestate is 2 × 10^14^ cells mol^−1^ O_2_, which has been used to calculate cell densities in the digestate for fertilization experiments. The cell densities are based on O_2_ consumption from a single vial, and technical replicates from that same vial were used for qPCR analysis (all points plotted). **Panel c and d:** The aerobic and anaerobic growth rates, culturing as explained for panel a. Panel c: O_2_-consumption rates, 6 vol% O_2_ in the headspace, inoculated with ~7 × 10^8^ cells mL^−1^, and estimated growth rate (*µ*, h^−1^) for each vial (average = 0.29 h^−1^, s.d. = 0.006). Panel d: rates of N_2_O reduction, anoxic vials with 1.1 vol% N_2_O in headspace, inoculated with ~3.4 × 10^8^ cells mL^−1^, and estimated *µ* (average = 0.11 h^−1^, s.d. = 0.001). Given these growth rates, and the growth yields (panel a) we calculate *V*_*max*_ = µ/Y): *V*_*maxN2O*_ = 0.65 fmol N_2_O cell^−1^ h^−1^, *V*_*maxO2*_ = 0.73 fmol O_2_ cell^−1^ h^−1^. The maximal electron transport rates are *V*_*e maxO2*_ = 2.9 fmol e^−^ to O_2_ cell^−1^ h^−1^, *V*_*e max e N2O*_ = 1.3 fmol e^−^ to N_2_O cell^−1^ h^−1^.

Based on the bioenergetics and charge separation for aerobic and anaerobic respiration of canonical denitrifying organisms, having NosZI (Box 1), the growth yield in terms of grams of cell dry weight per mole of electrons (${Y}_{e \mbox{-} {N}_{2}O}$) is about 60% of that for aerobic growth (${Y}_{e \mbox{-} {O}_{2}}$)^37^. For CB-01, which has NosZII, ${Y}_{e \mbox{-} {N}_{2}O}$ was 85% of ${Y}_{e \mbox{-} {O}_{2}}$ (Extended Data Fig. 1a,b), which lends support to the claim that electron flow to NosZII conserves more energy (by charge separation) than that to NosZI (refs. ^38,39^).

### Cell-specific respiration and growth rates

Measured aerobic and anaerobic respiration rates during unrestricted growth were used to estimate maximum growth rates, *µ*_max_, by nonlinear regression (Extended Data Fig. 1c,d), and the maximum rate of electron flow per cell to O_2_ and N_2_O was calculated on the basis of the measured growth yields (*V*_max_ = *µ*_max_/*Y*). The estimates are ${\mu }_{max{{\rm{O}}}_{2}}$ = 0.29 h^−1^ (s.d. = 0.006), ${\mu }_{max{{\rm{N}}}_{2}{\rm{O}}}$ = 0.11 h^−1^ (s.d. = 0.001), ${V}_{{{\rm{maxO}}}_{2}}$ = 0.72 fmol O_2_ per cell per hour, ${V}_{{{\rm{maxN}}}_{2}{\rm{O}}}$ = 0.66 fmol N_2_O per cell per hour. In terms of electron flow rates per cell, we get ${V}_{{{\rm{m}}{\rm{a}}{\rm{x}}{\rm{e}}-{\rm{O}}}_{2}}$ = 2.9 fmol of electrons to O_2_ per cell per hour, ${V}_{{{\rm{m}}{\rm{a}}{\rm{x}}{\rm{e}}-{\rm{N}}}_{2}{\rm{O}}}$ = 1.3 fmol of electrons to N_2_O per cell per hour. This shows that CB-01 slows down its respiratory metabolism by about 50% when switching from aerobic to anaerobic respiration.

### Oxygen repression of N_2_O respiration

**Extended Data Fig. 2:**
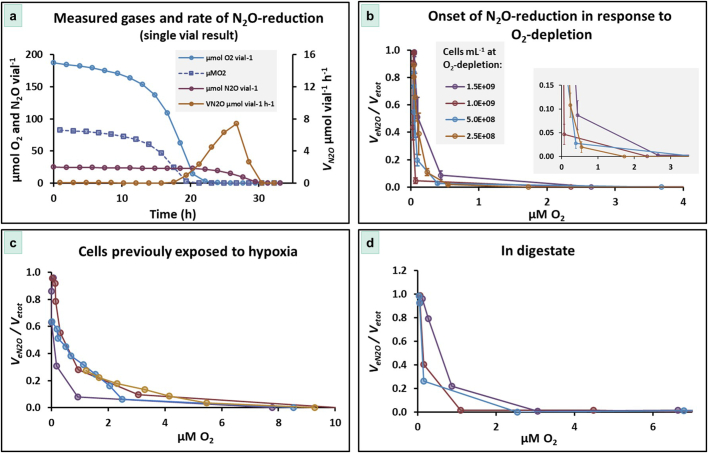
Onset of N_2_O-reduction during O_2_ depletion. Denitrifying bacteria vary as to how early they initiate anaerobic respiration during O_2_ depletion. To explore this for *Cloacibacterium* sp. CB-01, we ran several experiments, both in nutrient broth (panel a-c) and in digestate (panel d). **Panel a and b** show the results for experiments where the inoculum was raised through > 10 generations under strict oxic conditions, thus diluting out any N_2_O reductase that might be present in the cells. Panel a shows the gas measurements, the O_2_ concentration in the liquid as calculated from the O_2_ transport rate (see Molstad et al.^54^), and the rate of N_2_O-reduction (*V*_*N2O*_). Panel b shows the ratio *V*_*eN2O*_/*V*_*etot*_, i.e. the fraction of total electron flow that goes to N_2_O (for each time increment), plotted against the O_2_ concentration in the liquid. Error bars show s.d. of the mean for n = 3 replicate vials. **Panel c** shows *V*_*eN2O*_/V_etot_ (plotted against [O_2_]) for an experiment where the inoculum had been grown in hypoxia, thus with NosZ expressed already. **Panel d** shows the result for growth in digestate, inoculated with cells raised aerobically.

N_2_O respiration under oxic conditions has been reported for several organisms^31^. Such aerobic N_2_O respiration would be desirable for an organism to effectively scavenge N_2_O in soil, but we found no evidence for this in CB-01: aerobically raised cells monitored as they depleted oxygen did not initiate N_2_O respiration before the oxygen concentration reached below 1–2 µM, whereas cells previously exposed to anoxia (hence with intact NosZ enzymes) initiated N_2_O respiration at 4–6 µM O_2_ (Extended Data Fig. 2).

### Affinity for O_2_ and N_2_O

**Extended Data Fig. 3:**
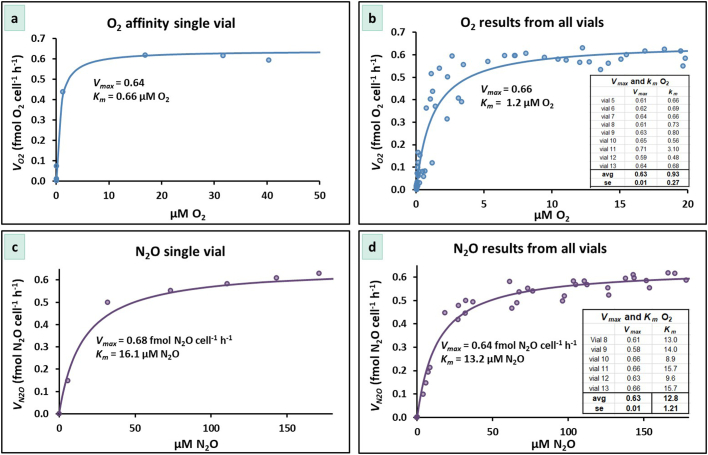
Apparent affinity for O_2_ and N_2_O. To assess the affinity for O_2_ and N_2_O, we measured the rates of O_2_- and N_2_O reduction as the batch cultures depleted the two electron acceptors. The rates as measured (mol vial^−1^ h^−1^) were converted to rates per actively respiring cell (*V*_*O2*_ and *V*_*N2O*_, fmol cell^−1^ h^−1^) based on the numbers of active cells in the vial at each time point (= the midpoint between two samplings). The number of active cells were the numbers of cells in the inoculum + new-grown cells as calculated from the cumulated consumption of O_2_ and N_2_O (and the growth yield per mol, Extended Data Fig. 1), as done previously for determining the affinity for NO^59^. For *V*_*N2O*_, the number of actively N_2_O-respiring cells was only a fraction of the total (as shown in Extended Data Fig. 4). The maximum rates *V*_*max*_ and the apparent *K*_*m*_ values were found by fitting the Michaelis Menten model V = *V*_*max*_ × S / (*K*_*m*_ + S) to the data (S is the concentration of O_2_ and N_2_O in the liquid) by least square, using the Generalized Reduced Gradient Solver in Excel. This was done for each individual vial, and for the collective datasets. **Panels a and b** show the results for O_2_, for a single vial (a) and for the entire dataset (b). Embedded in the panel is the estimated *K*_*m*_ for each individual vial. **Panels c and d** show the results for N_2_O, for a single vial (c) and for the entire dataset (d). Embedded in the panel is the estimated *K*_*m*_ for each individual vial. These results show a relatively strong affinity for O_2_ (*K*_*m*_ ~ 1 µM O_2_), and a rather weak affinity for N_2_O (*K*_*m*_ ~ 13 µM N_2_O).

It is commonly assumed that an organism’s ability to effectively mitigate N_2_O emissions depends on its affinity for N_2_O. We determined the apparent half-saturation constant for O_2_ and N_2_O reduction in CB-01 by nonlinear regression of rates per cell versus concentrations of the two gases in the liquid, and found ${K}_{{{\rm{mO}}}_{2}}$ = 0.9 µM O_2_ (s.e. = 0.27) and ${K}_{{{\rm{mN}}}_{2}{\rm{O}}}$ = 12.9 µM N_2_O (s.e. = 1.2; Extended Data Fig. 3). The relatively low ${K}_{{{\rm{mO}}}_{2}}$ was expected as the genome of CB-01 contains genes coding for cbb3-type high-affinity cytochrome *c* oxidases^8^.

### Comparing the N_2_O sink strength

**Fig. 1:**
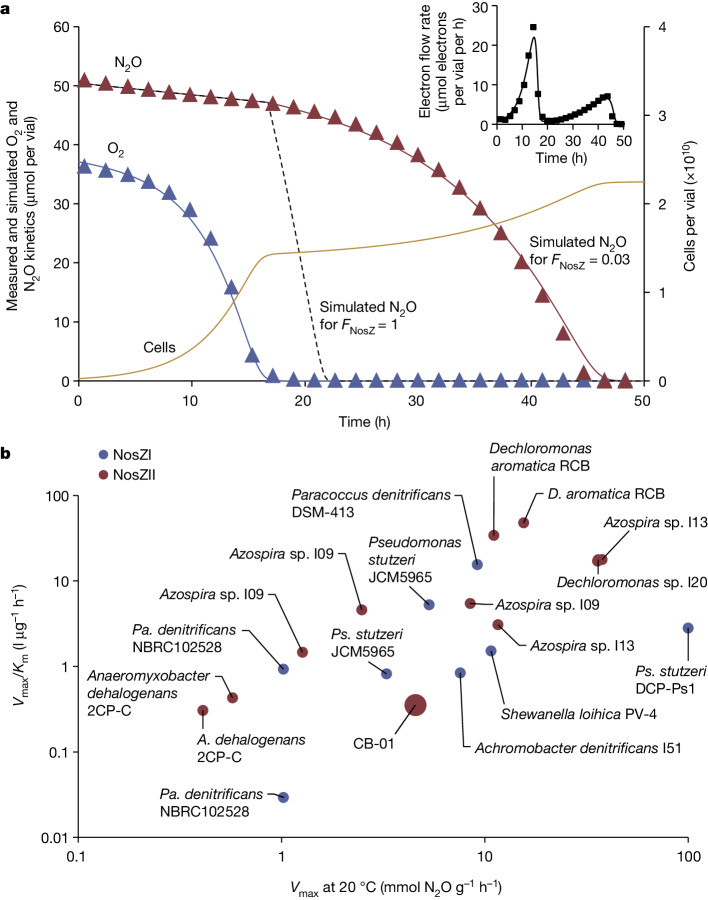
The biokinetics of N_2_O reduction, for CB-01 versus other strains. As judged by kinetics of N_2_O respiration in pure culture, CB-01 scores strikingly low compared to other N_2_O-respiring organisms as a sink for N_2_O: the kinetics of N_2_O respiration in response to O_2_ depletion indicate bet-hedging (that is, that only a fraction (*F*_NosZ_) of the cells express NosZ and start growing by N_2_O respiration after O_2_ depletion). **a**, The phenomenon for a single vial. Measured O_2_ and N_2_O (triangles), and simulated values (solid lines), using a simplified version of the bet-hedging model of ref. ^51^, with *F*_NosZ_ = 0.03. Of note, the decline of N_2_O concentrations before about 18 h is due to sampling loss. The yellow line shows the simulated cell density, and the dashed black line shows simulated N_2_O for *F*_NosZ_ = 1. The inset shows measured and simulated total electron flow in the vial. Two replicate vials showed very similar kinetics, and their *F*_NosZ_, estimated by model fitting, were 0.032 and 0.039. **b**, A condensed comparison of CB-01 with other N_2_O-respiring organisms regarding its capacity to scavenge N_2_O. Here we have plotted *V*_max_/*K*_m_ against *V*_max_ (mmol N_2_O per gram of cell dry weight per hour) for CB-01 and a range of other organisms with NosZI and NosZII, as measured by others (see Extended Data Table 1 for details and citations). The comparison shows that CB-01 is close to the average with respect to *V*_max_, but its *V*_max_/*K*_m_ ratio is very low owing to the low apparent affinity for N_2_O (*K*_m_ = 12.9 µM N_2_O).

**Extended Data Fig. 4:**
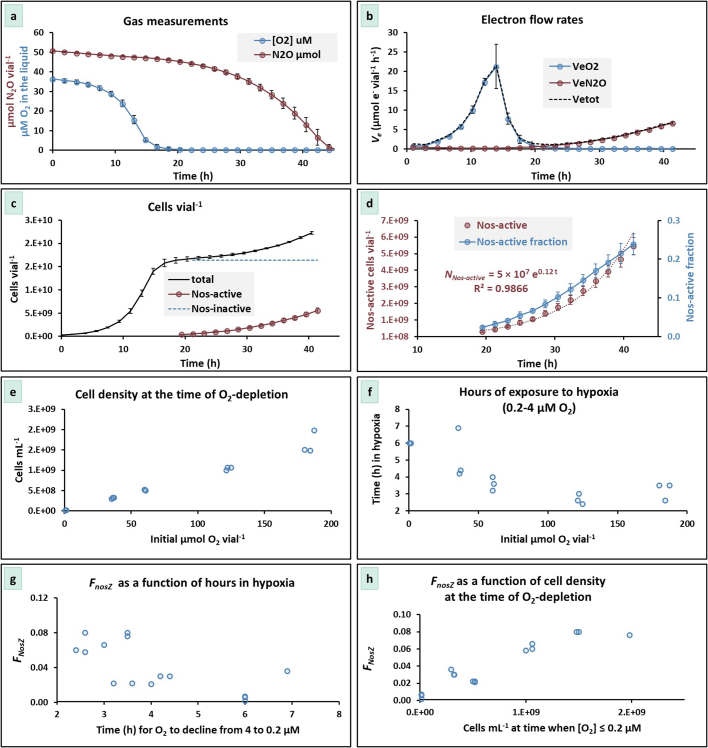
Bet-hedging during transition from aerobic to anaerobic respiration in CB-01. To investigate the characteristic denitrification regulatory phenotype of CB-01, 120 mL vials with 50 mL nutrient broth and O_2_ + N_2_O in He-atmosphere were inoculated with 2.7 × 10^8^ cells per vial, which had been raised under strict oxic conditions. The vials were monitored for gas kinetics as the cultures grew by oxygen initially, and then switched to respiring N_2_O in response to O_2_-depletion. The experiment included four treatments, all with 1 mL N_2_O (50 µmol N_2_O vial^−1^), but five different amounts of O_2_ (0, 0.8, 1.4, 3 and 4.6 mL O_2_) and 3 replicate vials for each O_2_ level. The panel shows the result for the vials with 0.8 mL initial O_2_. Error bars represent s.d. of the mean for n = 3 replicate vials, in all panels (4a-d). **Panel a** shows the measured amounts of O_2_ and N_2_O per vial. **Panel b** shows the electron flow rates to O_2_ and N_2_O (and total electron flow rate as a dashed line) as calculated from the measured O_2_ and N_2_O. Two phenomena stand out: 1) as oxygen was depleted, the electron flow rate declined to very low values and the subsequent electron flow rate to N_2_O increased exponentially, and 2) the apparent growth rate is 0.12 h^−1^, which is slightly higher than the anaerobic growth rate of CB-01 determined previously. This is the typical pattern for a bet-hedging denitrifying organism, i.e. an organism which expresses denitrification enzymes only in a fraction of the cells^32,51^. Assuming this, we investigated the possible fraction of cells that express NosZ and engaged in anaerobic respiration and growth: **Panel c** shows the estimated total number of cells (based on the cumulated O_2_- and N_2_O-consumption and the yields per mol O_2_ and N_2_O, Extended Data Fig. 1), and the number of N_2_O-respiring cells (Nos-active) calculated from the measured N_2_O-reduction rate (*V*_*N2O*_, mol N_2_O vial^−1^ h^−1^) and the assumption that *V*_*maxN2O*_ = 0.65 fmol N_2_O cell^−1^ h^−1^ (as determined previously): NosZ-active cells vial^−1^ = *V*_*N2O*_/*V*_*maxN2O*_. The blue dashed line is the estimated number of cells without Nos (Nos inactive), assumed to be cells entrapped in anoxia without NosZ, hence unable to synthesize NosZ. **Panel d** shows the number of Nos-active cells as numbers per vial, and as fraction of the total number of cells in the vial. This fraction increases with time due to growth by N_2_O-respiration, and the fraction at the time of O_2_ depletion is a crude estimate of the fraction of cells which were able to express NosZ before O_2_ is completely exhausted. We coin this fraction ***F***_***nosZ***_, analogous to ***F***_***den***_ for *Paracoccus denitfrificans*, which is the fraction of cells that express NirS before O_2_ is depleted^51^, thus avoiding entrapment in anoxia^61^. In *P. denitrificans*, ***F***_***den***_ was proportional to the time length of the hypoxic phase preceding complete anoxia, ascribed to a stochastic initiation of transcription of *nirS* once the cells experience hypoxia. To investigate if the apparent bet-hedging in CB-01 shows the same pattern, we estimated ***F***_***nosZ***_ for 15 batch cultures, all provided with 1 mL N_2_O but different amounts of O_2_ (0, 0.8, 1.4, 3 and 4.6 mL O_2_, n = 3 replicate vials for each O_2_-level). The cell density at the time of O_2_-depletion increased with increasing initial O_2_ (**panel e**), whereas the time length of exposure to hypoxia (arbitrarily defined as 0.2–4 µM O_2_) declined (**panel f**). A simplified version of the bet-hedging model^60^, assuming instantaneous expression of NosZ in a fraction of the cells (***F***_***NosZ***_) as O_2_ reached below 0.5 µM, was fitted to observed gas kinetics (O_2_ and N_2_O) for each vial to estimate ***F***_***NosZ***_ (all other parameters were as determined previously (Extended Data Figs. 1–2). Contrary to our expectations, the estimated ***F***_***NosZ***_ decreased with increasing time length of exposure to hypoxia (**panel g**) and increased with cell density at the time of O_2_ depletion (**panel h**). Interestingly, practically all cells appeared to become entrapped in anoxia (***F***_***NosZ***_ = 0.002-0.006) in the vials without any O_2_ injected. The results warrant further investigations to provide direct evidence for the cell differentiation (bet-hedging), and the mechanism causing ***F***_***NosZ***_ to increase with cell density. A tantalizing hypothesis is that quorum sensing induction of NosZ expression is involved. Interestingly, all cells expressed NosZ in response to oxygen depletion when growing in digestate (Extended Data Fig. 5d–g).

**Extended Data Fig. 5:**
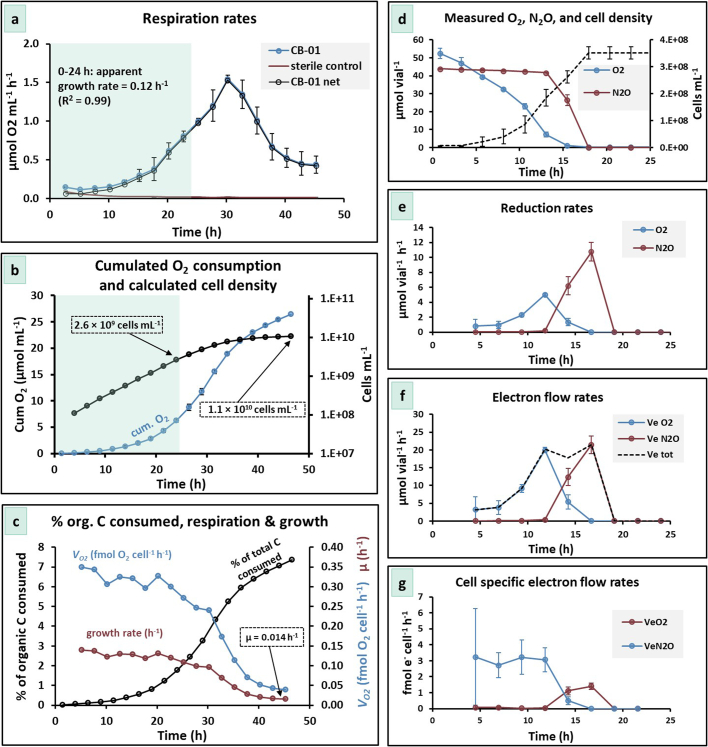
Aerobic respiration, growth and and transition anaerobic respiration of CB-01 in digestate. **Panels a-c** show the kinetics of O_2_-consumption during cultivation of CB-01 in digestate for the field experiments, measured in 50 mL subsamples placed in the incubation robot. The error bars (seen in all panels except c), represent the s.d. of the mean for n = 3 replicate vials. **Panel a** shows the rates of O_2_ consumption in vials with CB-01 and in the sterile controls, and the net consumption by CB-01 (CB-01 minus sterile control). This increased exponentially during the first 24 h, with apparent growth rate 0.12 h^−1^, which is much slower than in nutrient broth (0.29 h^−1^, Extended Data Fig. 1). **Panel b** shows the cumulated O_2_ consumption by CB-01, and the estimated cell density assuming *Y*_O2_ = 4.06 × 10^14^ cells mol^−1^ O_2_ (Extended Data Fig. 1), reaching 1.1 × 10^10^ mL^−1^. The cell density quantified by qPCR for a similar experiment only 47% of the density based on O_2_ (Extended Data Fig. 1), suggesting that *Y*_*O2*_ for growth to high cell densities in digestate is ~50% of *Y*_*O2*_ for optimal growth in nutrient broth (Extended Data Fig. 1b). **Panel c** shows estimated cell specific O_2_ consumption (*V*_*O2*_, fmol O_2_ cell^−1^ h^−1^), estimated growth rate, µ (h^−1^) = *V*_*O2*_ × *Y*, where *Y* = the measured growth yield by aerobic respiration (4.06 × 10^14^ cells mol^−1^ O_2_), and the estimated fraction of organic C in the digestate (10 mg C mL^−1^) consumed by CB-01 (sum of CO_2_ and assimilated C). This suggests that ~1 % of the organic C in the digestate was easily available monomers, supporting rapid growth of CB-01 to a cell density of ~1 × 10^9^ cells mL^−1^ after 20 h, while subsequent growth was gradually declining as the organism utilized increasingly recalcitrant substrates, plausibly with a lower growth yield (*Y*_*O2*_). **Panels d-g** show results of experiments designed to investigate if CB-01 is bet-hedging when growing in digestate: Bet-hedging of CB-01, as observed when cultured in nutrient broth (Extended Data Fig. 4) would reduce its capacity to scavenge N_2_O when vectored by digestate to soil. In theory, however, the bet-hedging could depend on the growth medium, since the fraction of cells expressing NosZ (***F***_***nosZ***_) increased with the cell density (Extended Data Fig. 4), plausibly due to accumulation of compounds stimulating the expression of NosZ. On this background, we conducted experiments similar to that shown in Extended Data Fig. 4, but using digestate instead of nutrient broth as a growth medium. The panels show the results of one treatment, in which 120 mL serum vials with 50 mL autoclaved and aerated digestate (and stirring magnets), He-atmosphere + 1.4 mL O_2_ and 1 mL N_2_O, were inoculated with 2.5 × 10^8^ CB-01-cells (5 × 10^6^ cells mL^−1^), raised under strict oxic conditions, and monitored for gas kinetics while incubated at 23 °C. **Panel d** shows the measured O_2_ and N_2_O, together with the cell density as calculated from the initial cell density and growth as calculated from the measured O_2_ and N_2_O-reduction, using the yield per mol O_2_ and N_2_O determined previously (*Y*_*O2*_ = 4 × 10^14^ cells mol^−1^ O_2_, *Y*_*N2O*_ = 1.7 × 10^14^ cells mol^−1^ N_2_O, Extended Data Fig. 1). **Panel e** shows the O_2_ and N_2_O consumption rates (µmol vial^−1^ h^−1^) for each time increment, and **panel f** shows the rates of electron flow to O_2_ (aerobic respiration) and N_2_O (anaerobic respiration), and the total electron flow as a dashed line. In contrast to the results with nutrient broth (Extended Data Fig. 4) there is hardly any depression of the electron flow in response to oxygen depletion, suggesting that ***F***_***nosZ***_ ~ 1, i.e. all cells switch to respiring N_2_O (express nosZ). To inspect the validity of this further, the electron flow rates per cell (fmol e^−^ cell^−1^ h^−1^) to O_2_ (*V*_*eO2*_) and N_2_O (*V*_*eN2O*_) were calculated, and shown in **panel g**. As expected, *V*_*eO2*_ remained stable around 3 fmol e^−^ cell^−1^ h^−1^ until oxygen became limiting, which is close to the maximum rate determined previously (*V*_*maxO2*_ = 0.72 fmol O_2_ cell^−1^ h^−1^ = 2.88 fmol e^−^ cell^−1^ h^−1^), and *V*_*eN2O*_ reached ~1.4 fmol e^−^ cell^−1^ h^−1^ immediately after O_2_-depletion, which is close to the maximum rate determined in nutrient broth cultures (*V*_*maxN2O*_ = 0.6 fmol N_2_O cell^−1^ h^−1^ = 1.2 fmol e^−^ cell^−1^ h^−1^). All panels show the average of three replicate vials, with standard deviation as vertical lines. The experiment included treatments with 0.8, 3 and 4.6 mL O_2_ (3 replicates of each), hence with widely different cell densities at the time of O_2_-depletion, and they all showed ***F***_***nosZ***_ to be close to 1 (results not shown). In summary, CB-01 express NosZ in all cells (hence no bet-hedging) in response to O_2_-depletion when grown in digestate, unaffected by the cell density at the time of O_2_-depletion.

To compare CB-01 with other organisms as a sink for N_2_O in soil, we have summarized the biokinetic parameters for various N_2_O-respiring organisms by plotting their ‘catalytic efficiency’ (*V*_max_/*K*_m_) against their *V*_max_ on a cell dry weight basis (Fig. 1b). This suggests that CB-01 is far from being the best among N_2_O-respiring organisms: it is on par with the average of others with respect to *V*_max_, which is a measure of the N_2_O sink strength at high N_2_O concentrations (»*K*_m_ = 12.9 µM N_2_O ≈ 389 ppmv in the gas phase at 15 °C), but it scores poorly at low N_2_O concentrations (*V*_max_/*K*_m_ for CB-01 is only 3% of the average for the others). The apparent bet-hedging (Fig. 1a), explored in more detail in several experiments (Extended Data Fig. 4) would clearly add to its inferiority as an N_2_O sink. However, the bet-hedging was clearly depending on the growth medium: when growing in digestate, all cells switch to anaerobic respiration in response to oxygen depletion (Extended Data Fig. 5d–g).

## Effects of CB-01 on N_2_O emissions

CB-01 was found to grow exponentially by aerobic respiration in autoclaved digestate, reaching a cell density of about 10^9^ cells per millilitre after 20 h. At this point, about 1% of the organic C in the digestate had been consumed, and the growth rate declined gradually, plausibly owing to depletion of the most easily available substrate components reaching a final density of about 6 × 10^9^ cells per millilitre after 2 days, as judged by oxygen consumption (Extended Data Fig. 5a–c), and growth yield based on quantitative PCR (qPCR) quantification of CB-01 cells (Extended Data Fig. 1b).

We conducted three outdoor experiments in which the soils were fertilized with digestates in which CB-01 had been grown to about 6 × 10^9^ cells per millilitre. Control treatments were fertilized with the same digestate, in which the CB-01 cells had been killed by heat (70 °C), thus securing practically identical N and C availability in the soils with and without metabolically active CB-01 cells. This type of control treatment is crucial for correctly assessing the effect of CB-01 metabolism, as the incorporation of any organic material will induce transient peaks of N_2_O emissions. Experimental details are provided in the Methods.

**Fig. 2:**
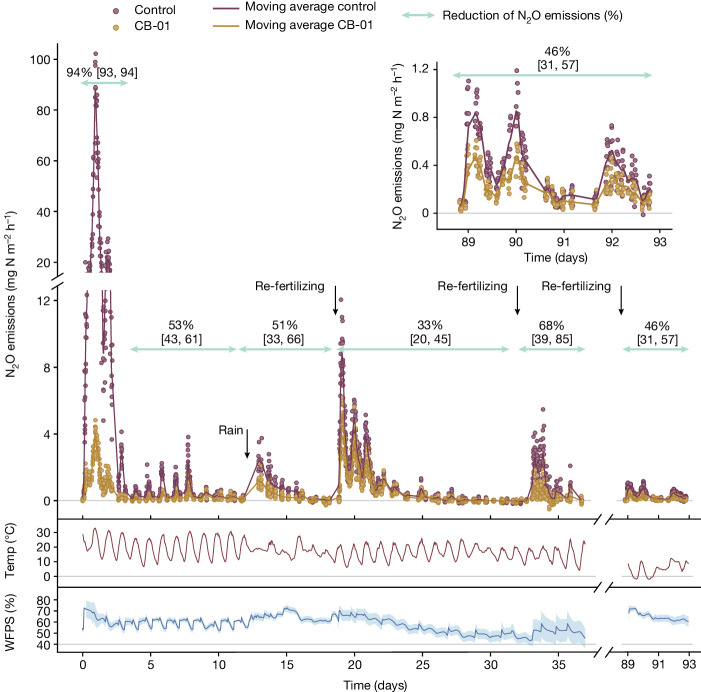
CB-01 effects on N_2_O emission from a clay loam soil of pH 6.7. N_2_O flux from buckets with soil throughout 90 days after fertilization (14 July 2021) with digestate (11 l m^−2^) in which the NNRB strain *Cloacibacterium* sp. CB-01 had been grown to about 6 × 10^9^ cells per millilitre, quantified by qPCR with primers specific for CB-01. Control buckets were fertilized with the same digestate in which CB-01 had been killed by heat (70 °C for 2 h). The buckets were sown with ryegrass (*Lolium perenne*), and the soil moisture content was sustained by daily water additions during the first 10 days. Buckets were re-fertilized with a lower dose of autoclaved and pH-adjusted digestate without CB-01 (4.6 l m^−2^) after 19, 33 and 89 days. The top panel shows N_2_O flux measured by the dynamic chamber method^52^ with 3 min enclosure time, operated by a field robot (Supplementary Fig. 1). The insert is a rescaled plot for day 89–93. The emissions are shown as single dots for each enclosure, and with a floating average for each treatment (solid lines, *n* = 8 replicate buckets for each treatment, calculated by a Gaussian kernel smoother). The lower panels show the average soil temperature (at 0–5.5 cm depth) and water-filled pore space (WFPS) from *n* = 4 loggers (s.d. of the mean is shown as lighter coloured ribbons). The fluxes show clear diurnal fluctuations, driven by temperature, and transient peaks in response to a rain event (day 12) and in response to re-fertilization (marked by arrows). The percentage reduction of N_2_O emissions (cumulated flux) by CB-01 was calculated for selected periods, shown by the green arrows with 95% confidential intervals (Methods). The additional control buckets receiving water instead of digestates emitted negligible amounts of N_2_O (result not shown).

The first field experiment demonstrated that the initial peak of N_2_O flux induced by the fertilization with digestate was practically eliminated by CB-01 (Fig. 2), and that CB-01 continued to have a strong effect throughout; a second peak in N_2_O emission induced by precipitation (day 12) was reduced by 51%; and the later emission peaks induced by re-fertilization with digestate without CB-01 (indicated by arrows) were reduced by 31, 67 and 46%.

Given the number of CB-01 cells added with the digestate (6.6 × 10^13^ cells per square metre of soil surface), and the *V*_max_ = 0.6 fmol N_2_O per cell per hour (Extended Data Fig. 1), the potential N_2_O consumption rate, if all the added CB-01 cells were respiring N_2_O at maximum rate, is 1.1 g N_2_O-N m^−2^ h^−1^. The peak N_2_O flux 1–2 days after fertilization was reduced by about 85 mg N_2_O-N m^−2^ h^−1^, which is about 8% of the estimated potential. For the subsequent peaks of N_2_O flux, the apparent N_2_O respiration by CB-01 (that is, the reduction of the flux) was ≤4 mg N_2_O-N m^−2^ h^−1^, which is ≤0.36% of the initial potential. This decline in apparent N_2_O respiration by CB-01 was plausibly a result of two factors: a gradually declining rate of N_2_O provision by the indigenous microbiome, and a gradually declining number of CB-01 cells.

**Fig. 3:**
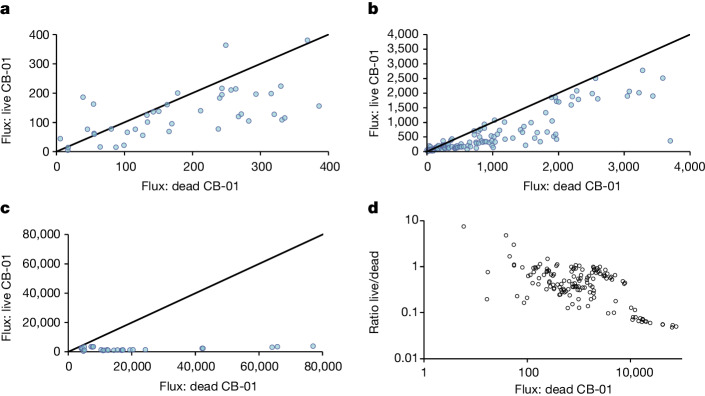
Inspecting the contingent effect of CB-01. Emissions from soil fertilized with digestate containing live *Cloacibacterium* sp. CB-01 plotted against the emissions from soil fertilized with digestate containing dead CB-01 cells (same data as in Fig. 2). **a**–**c**, The results for the low-emission (<400 μg N_2_O-N m^−2^ h^−1^; part **a**), intermediate-emission (<4,000 μg N_2_O-N m^−2^ h^−1^; part **b**) and high-emission (>4,000 μg N_2_O-N m^−2^ h^−1^; part **c**) ranges. **d**, A log-scaled plot of the ratio between emissions from soil with live and dead CB-01 plotted against the emission from soil fertilized with digestate containing dead CB-01.

One would expect that the effect of CB-01 as an N_2_O sink would be marginal in periods with low emissions: low emissions are due to low water-filled pore space (that is, drained soil), low respiration rate (limited by available organic C substrates) or both, resulting in marginal hypoxic and anoxic volumes within the soil matrix^40^. Under such conditions, the primary source of N_2_O emission could be nitrification^41^, and CB-01 as an N_2_O sink would be confined to the remaining hypoxic microsites. Inspections of the relationship between the effect of CB-01 and the N_2_O emissions in the control soil (that is, with dead CB-01) lend some support to this: although CB-01 reduced the emissions even for periods with modest emissions, the effect was clearly strongest in periods with high emissions (Fig. 3).

**Fig. 4:**
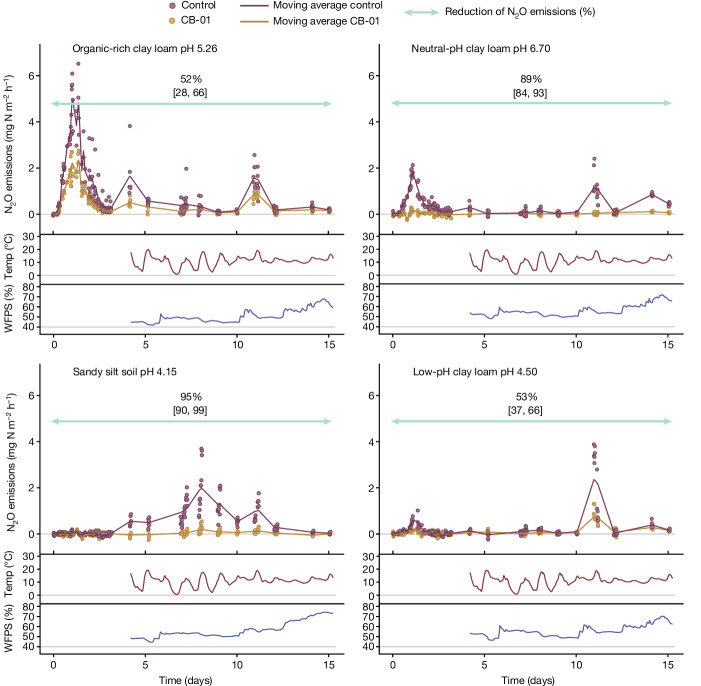
Reduction of N_2_O emissions in different soils in field buckets. The measured emission after application of digestates with and without CB-01 to four different soils (17 September 2021). The organic carbon contents of the soils were 15.8% (organic-rich clay loam of pH 5.26), 3.21% (neutral-pH clay loam of pH 6.70), 0.75% (sandy silt soil of pH 4.15) and 3.23% (low-pH clay loam of pH 4.50) of dry weight. The pH(CaCl_2_) before fertilization with digestate is given in the panels. The emissions are shown as single dots for each enclosure, and with a floating average for each treatment (solid lines, *n* = 6 replicate buckets for each treatment) as in Fig. 2. The percentage reduction of N_2_O emissions (cumulated flux) by CB-01 is shown by the green arrows with 95% confidential intervals (Methods).

**Extended Data Fig. 6:**
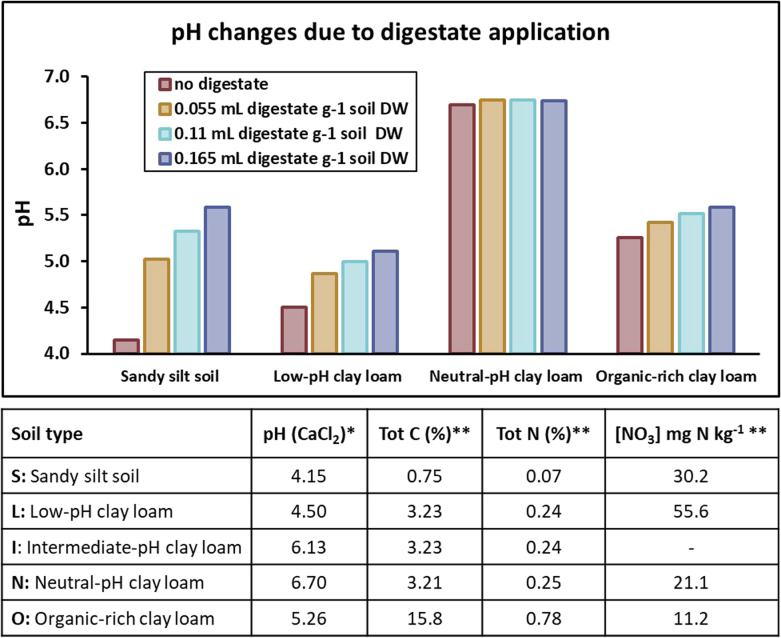
Soil characteristics and changes to soil pH due to application of digestate. Some key characteristics of the different soil types are listed in the table above. The acid sandy silt soil (**S**) was taken from an agricultural field in Solør, Norway, dominated by fluvial sandy silt soils. The clay loam soils **L**, **I** and **N** were from different plots within a liming experiment near the Norwegian University of Life Sciences (59°39’48.2”N 10°45’44.8”E), limed in 2014^41^. **O** was a clay loam soil from the same area (hence with similar mineral components), but with a much higher content of organic C because it had been a wetland prior to cultivation. Soils **S**, **L**, **N** and **O** were used in the field bucket experiments. Soil **I** is the soil of the plots used for the field plot experiment. The bar chart shows pH(CaCl_2_) in the four soils as affected by applying 0.055, 0.11 and 0.165 mL digestate g^−1^ soil dry weight, which is 50, 100 and 150 % of the amounts added to the soils in the field bucket experiment (0.11 mL digestate g^−1^ soil dry weight). Water was added (if needed) to reach a water-filled pore space (%) equivalent to that in the buckets after digestate application. *pH(CaCl_2_) was measured after dispersing 10 g soil in 25 mL of 0.01 M CaCl_2_. **Tot C = total organic C, Tot N = total organic N, and [NO_3_] = mg NO_3_N kg^−1^ soil dry weight.

We reasoned that the capacity of CB-01 to reduce N_2_O emissions could be influenced by soil type. Soil pH is plausibly crucial because the synthesis of functional N_2_O reductase is increasingly impeded by declining pH within the range 4–7, both in CB-01 (ref. ^7^) and most other NRB^5^. Soil organic carbon content (SOC) could also have an impact. This is because the abundance of CB-01 relative to the abundance of indigenous N_2_O-producing bacteria would be inversely related to SOC, as the abundance of indigenous bacteria in soil is directly related to SOC^42^. To explore this, we replicated the bucket experiment (Fig. 2), but with four different soils spanning a range of pH levels and including a soil with very high organic carbon content (Fig. 4 and Extended Data Fig. 6).

The emissions were low compared to those in the first experiment, plausibly owing to lower temperatures (September versus July), but CB-01 significantly reduced the emissions from all four soils. The strong effect in the acidic sandy silt soil (pH 4.15) was unexpected, as CB-01 proved unable to reduce N_2_O at such low pH (ref. ^7^). However, the incorporation of digestate in this soil increased the pH(CaCl_2_) of the sandy silt soil by more than one pH unit (Extended Data Fig. 6), reflecting its weak buffer capacity. Most probably, the CB-01 embedded in the digestate experienced an even higher local pH (pH of the digestate was 7.3). The results for the three clay loam soils show a stronger effect of soil pH: CB-01 had a clearly stronger effect in the neutral-pH clay loam (pH 6.7) than in the two more acidic clay loams (low-pH clay loam of pH 4.5; organic-rich clay loam of pH 5.26).

**Fig. 5:**
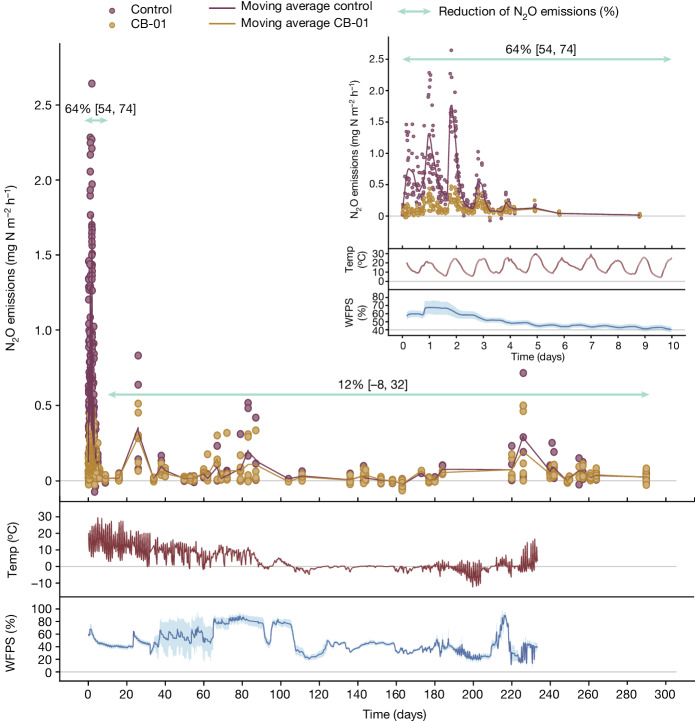
Reduction of N_2_O emissions in field plots. The 0.5-m^2^ field plots with clay loam of pH 6.13 were fertilized by mixing digestate into the upper 10 cm (20 August 2022), with live and dead CB-01 as in previous experiments (*n* = 6 replicate plots for each treatment). The top panel shows emissions throughout 290 days, and the insert shows emissions during the first 10 days. The percentage reduction of N_2_O emissions (cumulated flux) by CB-01 for the periods 0−10 and 10–290 days is shown by the green arrows with 95% confidential intervals (Methods). The lower panels show soil temperature and WFPS for *n* = 4 loggers, with ribbons representing the s.d. of the mean.

Finally, we scaled up to a field plot experiment, fertilizing 0.5-m^2^ plots with digestate with live and dead CB-01, mixed into the upper 10-cm layer of the soil as in the bucket experiments. The experiment was conducted on field plots that had been limed with 2.3 kg m^−2^ of dolomite in 2014, with an average pH(CaCl_2_) = 6.13 (s.d. = 0.10). The high emissions during the first 4 days (Fig. 5) show diurnal variations, peaking when the soil temperatures reach their maximum, and a substantial effect of CB-01. Subsequent emissions, measured at low frequency throughout 280 days, were much lower and the effect of CB-01 was not statistically significant, albeit with a wide confidence interval. The very low soil temperature could be the reason for the meagre effect.

## Survival in soil

Soil microbiome engineering by inoculation is an emerging field, promising new possibilities in enhancing agricultural efficiency and sustainability^43^. It is challenging, however, because inoculants are invariably found to die out rapidly, plausibly due to a multitude of abiotic and biotic barriers impeding establishment^44^. CB-01 was obtained through a dual substrate enrichment technique aimed at isolating organisms capable of withstanding the abiotic challenges of soil^7^. However, this selection process did not account for the biotic barriers that organisms may encounter in soils, such as competition for resources, antagonism and predation, as highlighted previously^45^.

**Fig. 6:**
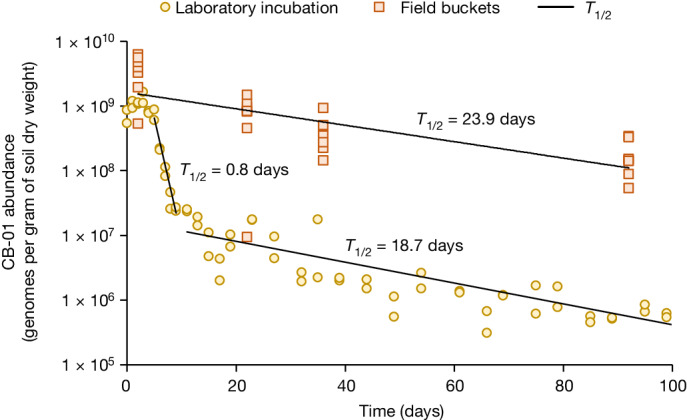
Survival of CB-01 in soil. The abundance of CB-01 was assessed by qPCR (Methods). The panel shows the genome abundance in the long-term field bucket experiment (Fig. 2) and in the laboratory incubation experiment (Methods). In the field bucket experiment, additional digestate (without CB-01) was incorporated 2 days before each soil sampling for qPCR. A single dot represents an individual soil sample (*n* = 8), and the line is the fitted exponential function *N*_*t*_ = *N*_0_e^−*d*×*t*^, in which *N*_*t*_ is the abundance at time *t*, and *d* is the apparent first-order death rate (estimated half-life *T*_1/2_ = ln(2)/*d*). For the laboratory incubation, three phases can be recognized: an initial apparent growth during the first 2–3 days, followed by a rapid first-order decline during the subsequent 4–5 days, and a slow first-order decline thereafter. Of note, the measured CB-01 genome abundance in the field plots after 280 days indicated similar average first-order death rates (0.02 per day, *T*_1/2_ = 34 days; Extended Data Table 2).

To assess the ability of CB-01 to survive in soil, we used qPCR with specific primers to measure the abundance of CB-01 genomes in soil (Methods) throughout the long-term field bucket experiment (Fig. 2), and throughout a laboratory incubation of soil amended with digestate with CB-01 (Methods); the results are shown in Fig. 6. During the laboratory incubation, there was a fast first-order reduction in abundance during days 3–7, and a much slower first-order reduction thereafter. By contrast, the abundance was sustained at a high level throughout 90 days in the field buckets, albeit gradually declining. The sustained CB-01 population in the bucket experiment explains why the effect on the N_2_O emission was sustained (Fig. 2).

The discrepancy between the field and the laboratory experiments demands a scrutiny. In the field bucket experiment, digestate (not inoculated with CB-01) was applied three times during the course of the experiment, with soil sampling for quantification of CB-01 abundance conducted 2 days after each application. As digestate is a suitable substrate for CB-01, growth of CB-01 in response to each dose could contribute to the sustained population.

Another factor could be protozoal grazing, which was plausibly more intense in the laboratory incubations than in the field experiment, owing to the higher soil moisture content at the time of CB-01 incorporation. In the laboratory experiment, the digestate with CB-01 was dripped onto soil that was already very wet (0.53 ml per gram of soil dry weight) and retained this high soil moisture throughout. In the field bucket experiment, CB-01–digestate was harrowed into relatively dry soil (0.34 ml per gram of soil dry weight), and the soil remained modestly moist throughout (Fig. 2). There is ample evidence that low soil moisture protects a bacterial inoculum against protozoal grazing, ascribed to increasing tortuosity, and localization of bacteria in small pores that are inaccessible to the protozoa^46^. Although we recognize that this is a speculative explanation, it warrants further experimental investigation owing to the potential practical implications.

**Extended Data Fig. 7:**
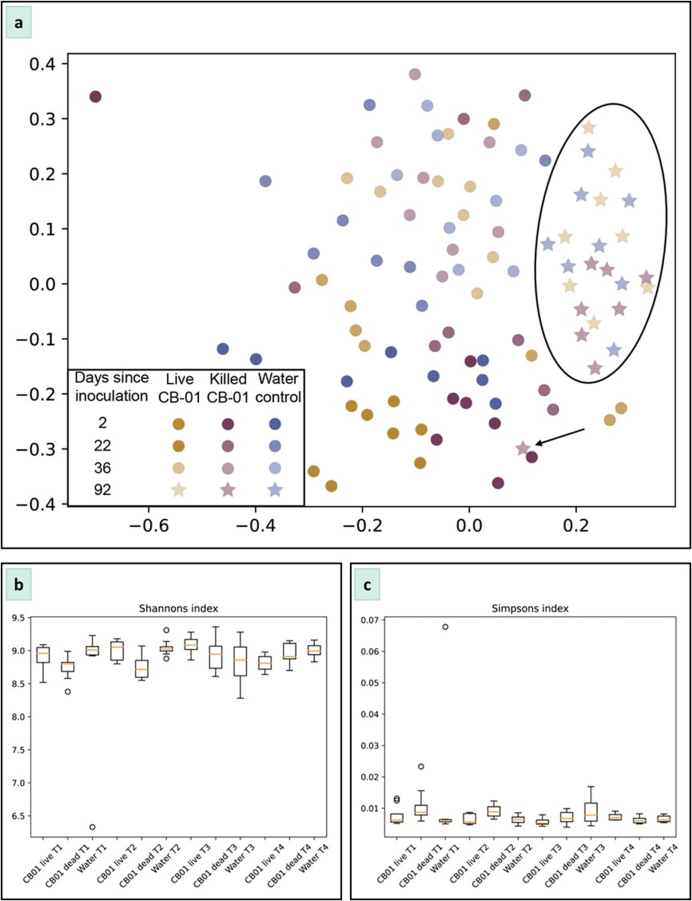
The influence of CB-01 inoculation on the soil microbial communities. **Panel a** shows an MDS plot based on Jaccard’s dissimilarity measures for microbial communities in the soil of the first field bucket experiment treated with live CB-01 in digestate, killed CB-01 in digestate, and water treatment only (Methods 8). The panels show the results for soils sampled 2, 22, 36 and 92 days after fertilization with digestate containing CB-01. Results for the last sampling (92 days) are encircled, and a single outlier is marked by arrow. **Panel b** shows the Shannon’s diversity indices for microbial soil communities sampled throughout the field bucket experiment treated with live CB-01 in digestate, killed CB-01 in digestate and water treatment only. **Panel c** shows the Simpson’s diversity indices for microbial soil communities sampled throughout the field bucket experiment treated with live CB-01 in digestate, killed CB-01 in digestate and water treatment only. Boxes in the box plots shown in panels b and c indicate the interquartile range with a line within the box representing the median. The whiskers extending from the box extend to the furthest datapoint contained within 1.5 times the interquartile range from the boxes and circles past these whiskers denote outlier values (n = 8).

A legitimate concern would be that the heavy inoculation with CB-01 could affect the indigenous microbiota^47^. We investigated this by analysis of 16S rRNA gene amplicons, excluding the operational taxonomic unit that circumscribed CB-01 (Methods), and found that the digestate itself had a transient impact (with or without live CB-01), but we were unable to discern any consistent difference between the treatments with live versus dead CB-01, which both converged towards the composition of pristine soil (Extended Data Fig. 7).

Laws and regulations for the use of inoculants vary from country to country, but all are likely to forbid the use of NNRB if they carry genes for antibiotic resistance or pathogenicity. We were unable to identify such genes in CB-01 (Methods).

## Extrapolating to national emissions

To assess the potential emission reductions by NNRB compared with other available techniques such as optimized N fertilization and nitrification inhibitors, we estimated emissions for Europe 2030 with the greenhouse gas and air pollution interactions and synergies (GAINS) model^48,49^ (Methods).

Consistent with using a uniform emission factor in GAINS (from the Intergovernmental Panel on Climate Change (IPCC)^50^) of 1% of N applied to be emitted as N_2_O, a uniform factor for emission reductions was also assumed. From the experiments, we conclude that 60% of emission reductions due to NNRB may be considered a conservative estimate. In Extended Data Table 3, emission reductions are shown by European country for a 2030 scenario if emissions from the application of liquid manure alone are reduced by 60%. All other anthropogenic emissions have been left unchanged. Under these assumptions, the total anthropogenic N_2_O emissions from Europe decrease by 2.7% owing to NNRB being introduced and applied to all liquid manure systems. This figure is higher in countries that have a high share of liquid manure systems in their agriculture; hence, it increases to 4.0% for EU27 (27 EU member countries).

Ongoing work explores the possibility to extend the technology by growing NNRB in all types of organic waste used to fertilize soils, and by combining the application of mineral N fertilizers with incorporation of NNRB-amended organic wastes. This requires new strains, technologies and investments, but with a great potential, reducing EU27 agricultural emissions by a third (31%; Extended Data Table 3).

It needs to be pointed out that an emission reduction of 60% as derived here for NNRB is much larger than emission reductions typically reported for N_2_O abatement measures. GAINS, for example, assumes nitrification inhibitors to be able to reduce emissions by as much as 38%, and high-tech mechanical fertilizer-saving technologies (‘variable rate application’) to be able to save only 24% of the emissions^48^.

## Future development

This study presents a proof of concept demonstrating a feasible utilization of NNRB to curb N_2_O emissions from farmland. By using organic waste as substrates and vectors, massive soil inoculation is achieved, which can secure reduced N_2_O emissions throughout an entire growth season, despite a gradually declining NNRB abundance. To ensure the robustness and versatility of this biotechnology, we will need an ensemble of new NNRB strains, capable of thriving in waste materials beyond digestates. New NNRB strains will probably vary regarding their ability to tolerate abiotic and biotic stress factors present in the soil. The dual substrate enrichment technique^7^ selects for strains tolerant of abiotic, but not biotic, stress. Consequently, innovative techniques are necessary for selecting strains that tolerate the biotic stress.

## Methods

### Robotized batch cultivations for respiratory phenotype

NNRB have attracted much interest recently as net sinks for N_2_O in soils, potentially curbing N_2_O emissions^4,31^. NNRB strains vary grossly in their apparent capacity to act as N_2_O sinks, assessed by determining their biokinetic parameters: NNRB strains are commonly assumed to be strong N_2_O sinks if they have strong affinity (low apparent *K*_m_) for N_2_O and a high maximal rate of N_2_O reduction (*V*_max_), or simply a high catalytic efficiency (that is, a high *V*_max_/*K*_m_)^38^. Another desirable, albeit speculative, feature would be to reduce N_2_O under oxic or at least hypoxic conditions^53^.

To assess *Cloacibacterium* sp. CB-01 along these criteria, we conducted in-depth investigations of its respiratory phenotype by batch culturing in the robotized incubation system designed and described previously^54,55^, with the OpenLAB CDS 2.3 software for GC data acquisition (Agilent). The system hosts up to 30 parallel stirred batch cultures (normally 50 ml) in 120-ml gas-tight serum vials (crimp-sealed with butyl rubber septa) with a He atmosphere (with or without N_2_O and O_2_), which are sampled frequently for measuring the concentrations of O_2_, N_2_, N_2_O, NO and CO_2_ in the headspace. Robust routines are established for calculating the rates of production and consumption of all the gases (taking sampling loss and leakage into account), and for calculating gas concentrations in the liquid as a function of measured gas concentrations in the headspace and the rate of transport between liquid and headspace. These routines are included in a spreadsheet that is publicly available, including a set of instruction videos^56^. The system has been used in numerous investigations of the respiratory phenotypes of denitrifying bacteria^6,7,33,57–62^.

To enable refined analyses of the respiratory phenotype of CB-01, we initially determined the cell dry weight (femtograms per cell), and the growth yields for aerobic (${Y}_{{{\rm{O}}}_{2}}$, cells per mole of O_2_) and anaerobic (${Y}_{{{\rm{N}}}_{2}{\rm{O}}}$, cells per mole of N_2_O) respiration by measuring the cell yields in batches provided with various amounts of O_2_ and N_2_O. This enabled inspection of the cell-specific respiration rates (fmoles per cell per hour) throughout subsequent batch incubations, based on measured rates (moles of O_2_ and N_2_O per vial per hour) for each time interval between two gas samplings, and the estimated cell number in the vial for the same time interval (=*N*_ini_ + ${Y}_{{{\rm{O}}}_{2}}$ × cumO_2_ + ${Y}_{{{\rm{N}}}_{2}{\rm{O}}}$ × cumN_2_O, in which *N*_ini_ is the initial number of cells at time 0, and cumO_2_ and cumN_2_O are the cumulated consumption of the two gases). The cell-specific rates calculated this way allowed an analysis of the affinity for O_2_ and N_2_O by plotting cell-specific rates of O_2_ and N_2_O against the concentrations of the two gases in the liquid as the cultures depleted the gases, and fitting the Michaelis–Menton function to these data (least squares). Batch cultures provided with both N_2_O and O_2_ in the headspace were monitored as they depleted O_2_ and switched to respiring N_2_O, thus determining the critical concentration of O_2_ (in the liquid) at which the cells started to respire N_2_O. The kinetics of electron flow throughout such transitions from aerobic to anaerobic respiration were used to assess the fraction of cells expressing N_2_O reductase in response to O_2_ depletion, using a simplified version of the model developed previously^60^.

All phenotype experiments were conducted at 23 °C. The medium used was GranuCult nutrient broth (product number 1.05443, Merck): 8 g l^−1^, containing meat peptone and meat extract, pH-adjusted to 7.3 with NaOH. Additional experiments were conducted with autoclaved digestate (aerated and pH-adjusted to 7.3, as described below).

### Culturing CB-01 in digestate for field experiments

For each field experiment, fresh digestate was collected from a wastewater treatment plant close to Oslo (VEAS), described in ref. ^6^. Averaged values of the quality parameters for the period of digestate collection were: dry matter content = 3.97 wt% (s.d. = 0.16), ignition loss of dry matter = 55.6% (s.d. = 2), pH = 7.72 (s.d. = 0.07) and NH_3_ + NH_4_^+^ = 1.71 g N l^−1^ (s.d. = 0.12).

Before cultivation of CB-01, the digestate was heat-treated, aerated and pH-adjusted. For the field bucket experiments, the digestate was autoclaved (121 °C for 20 min), and then sparged with air (while stirred) for 48 h to secure chemical oxidation of Fe^2+^ to Fe^3+^, and then autoclaved again. Oxidation of Fe^2+^ by air sparging was considered necessary to avoid abiotic oxygen consumption, as the digestate had high concentrations of Fe^2+^ originating from the Fe^3+^ used as precipitation chemicals in the primary wastewater treatment, and reduced to Fe^2+^ in the anaerobic digesters^6^. The sparging caused the pH to increase to 9.4 owing to the removal of CO_2_, requiring a final pH adjustment to 7.3 (with HCl). The same procedure was used for the field plot experiment, except that autoclaving was replaced by heat treatment: 70 °C for 4 h.

CB-01 was then grown aerobically in the pretreated digestates, inoculated to an initial cell density of about 5 × 10^7^ cells per millilitre, which were stirred and sparged with sterile air (filtered) at 23 °C. To monitor the growth of CB-01, we transferred subsamples of each batch (after inoculation) to 120-ml vials (50 ml per vial) with Teflon-coated magnetic stirring bars, which were placed in the incubation robot system for monitoring the O_2_ consumption (Extended Data Fig. 5a–c).

### Field experiments

Emissions of N_2_O in all outdoor experiments were monitored by the ‘dynamic chamber’ technique^52,63^, operated by an autonomous field flux robot described previously^64^, and shown in detail in Supplementary Fig. 1.

#### Field bucket experiments

Soils for the bucket experiments were collected from agricultural fields in southern Norway, spanning a range of soil characteristics. The acid sandy silt soil (S) was taken from an agricultural field in Solør, Norway, dominated by fluvial sandy silt soils. The clay loam soils L, I and N were from different plots within a liming experiment near the Norwegian University of Life Sciences (59° 39′ 48.2″ N 10° 45′ 44.8″ E), limed in 2014 (ref. ^41^): the low-pH clay loam (L) received no lime, the intermediate-pH clay loam (I) was limed with 2.3 kg m^−2^ of dolomite, and the neutral-pH clay loam (N) was limed with 3 kg m^−2^ of finely ground calcite. Soil O was a clay loam soil from the same area as L, I and N (hence, with similar mineral components), but with a much higher content of organic C because it had been a wetland before cultivation. The soil characteristics are listed in Extended Data Fig. 6.

The soils used in the bucket experiments (S, L, N and O) were sieved (10 mm) in moist conditions and mixed thoroughly before filling into the buckets. The conically shaped buckets (height = 21.5 cm, top diameter = 23.5 cm, bottom diameter = 21.5 cm) had a total volume of 8.6 l. An approximately 1-cm layer of gravel (4–8 mm diameter) was placed at the bottom, covered with a nylon fibre cloth to prevent eluviation of the soil by drainage. For soils S, L and N, 8 kg soil dry weight was filled into each bucket, packed by thumping the bucket on the ground until the soil had reached a bulk density of 1 kg l^−1^. For the organic-rich clay loam soil, each bucket was filled with only 5.92 kg soil dry weight, reaching a bulk density of 0.74 kg l^−1^ after being packed to 8 l. The soil surface area of the buckets was 0.043 m^2^.

To secure equal initial amounts of NO_3_ m^−2^ for all soils, we mixed an amount of KNO_3_ to each soil to reach a level of 12 g N m^−2^ soil surface = 516 mg NO_3_-N per bucket (soil surface area = 0.043 m^2^). Digestate (480-ml per bucket = 11 l m^−2^ soil surface area) was mixed into the top ≈10 cm of the soil by ‘harrowing’, using a small hand-held rake. We used autoclaved digestates in which CB-01 had been grown to about 6 × 10^9^ cells per millilitre, and as the control treatment we heat-treated this digestate (70 °C, 2 h), which effectively killed the CB-01 cells (tested by measuring respiration, results not shown). As an additional control treatment, buckets received water alone. The density of CB-01 cells per soil surface area immediately after application was 6.6 × 10^13^ cells m^−2^. The cell density in the upper 10 cm of the soil was about 6 × 10^8^ cells per gram of soil dry weight for the soils S, L and N (bulk density = 1 kg l^−1^), and about 8 × 10^8^ g^−1^ for soil O.

The buckets were placed on 1-m^2^ Plexiglass plates (1.5 mm), to avoid gas exchange with the soil below. The soil moisture (volumetric water content, m^3^ m^−3^) and temperature (°C) in the upper 5.5 cm of the soil were monitored by four Teros 11 sensors, connected to an EM50 logger (Meter Group). Emissions were measured by field flux robot, lowering the chambers over the buckets (Supplementary Fig. 1g).

In the first bucket experiment, using only soil N (Extended Data Fig. 6), starting on 14 July 2021, ryegrass (*L.* *perenne*) was sown the day after the incorporation of the digestate, and the emissions were monitored for 90 days. Within this time span, we added 200 ml autoclaved and pH-adjusted digestate (4.6 l m^−2^) without CB-01 three times (after 19, 33 and 89 days), to induce transient bursts of N_2_O emission. By the end of each burst of N_2_O emission induced by applying digestates, the upper 10 cm of the soil was sampled with an auger (diameter 1 cm) and stored in the freezer (−4 °C) until DNA extraction and subsequent molecular work. The auger was washed and sterilized with 70% ethanol between each sampling.

In a follow-up bucket experiment, all soils were included and monitored for 10 days, with no re-fertilization. Soil sampling was carried out after the first peak of N_2_O emissions, as described for the 90-day bucket experiment.

The digestate application’s influence on soil pH was tested in the laboratory by mixing soil with the same type and amount of digestate as applied to the 0–10-cm soil layers of the field buckets (0.11 ml per gram of soil) ±50% to show the potential pH in pockets with higher or lower than average concentration of digestate. Water was added (if needed) together with digestate to reach the same water-filled pore space (%) as in the field bucket experiment. The most prominent increase in soil pH was seen in the sandy silt soil (Extended Data Fig. 6), reflecting its low buffer capacity due to low content of clay and organic material (Extended Data Fig. 6), both known to be crucial factors determining the buffer capacity of soil^65^.

#### Field plot experiment

We established small (0.5 m^2^) test plots within larger field plots (8 m × 3 m) of a soil liming experiment (limed in 2014) on clay loam soil^41,66^ and re-limed with 174 g dolomite per square metre in 2019. We used the plots with soil I (Extended Data Fig. 6) that were previously limed with dolomite to pH(CaCl_2_) = 6.13 (s.d. = 0.10), and within each of the six replicate plots, we established two 0.7 m × 0.7 m test plots side by side (distance = 30 cm), fertilized with autoclaved digestate in which CB-01 had been grown to a cell density of about 6 × 10^9^ cells per millilitre. We applied 4.5 l digestate per plot (= 9 l m^−2^), which was mixed into the upper ≈10 cm of the soil by a hand-held cultivator. The initial density of CB-01 was 5.4 × 10^13^ cells per square metre. If distributed throughout the soil layer that was sampled for analyses (0–10 cm depth = 125 kg soil dry weight per square metre, assuming a bulk density of 1.25 kg l^−1^), the initial cell density in the soil would be 4.3 × 10^8^ cells per gram of soil. Soil samples for determining CB-01 abundance were taken from each plot (three replicate samples) before incorporation of digestate with CB-01, 9 days later, and after 10 months. The soil samples were stored in the freezer (−20 °C) until DNA extraction and following quantification by PCR.

The 0.5-m^2^ test plots were situated along the boardwalk for the autonomous field flux robot, which was used to monitor the N_2_O emissions (Supplementary Fig. 1f).

### Calculations of emissions and statistical analyses

From the slope of the N_2_O regression lines (Supplementary Fig. 1e), the flux of N_2_O is calculated by the equation

$${q}_{{{\rm{N}}}_{2}{\rm{O}}}=\frac{{10}^{-6}\,ahp}{RT}$$

in which ${q}_{{{\rm{N}}}_{2}{\rm{O}}}$ is the flux of N_2_O (mol m^−2^ s^−1^), *a* is the slope of the regression line (ppm s^−1^), *h* is the height (that is, the volume divided by the ground surface area) of the chamber (m), *p* is the pressure (Pa), *R* is the universal gas constant (J mol^−1^ K^−1^) and *T* is the temperature (K).

For graphic presentation of the emissions, we used the Gaussian kernel smoother^67^ to plot floating averages for each treatment (solid curves) together with individual measurements (as dots; Figs. 2, 4 and 5).

Cumulated N_2_O emissions over a period of time are approximated by using the trapezoidal rule on the estimated fluxes $(\int {q}_{{{\rm{N}}}_{2}{\rm{O}}}(t){\rm{d}}t\approx \sum ({q}_{{{\rm{N}}}_{2}{\rm{O}}}\left({t}_{i}\right)+{q}_{{{\rm{N}}}_{2}{\rm{O}}}\left({t}_{i+1}\right))({t}_{i+1}-{t}_{i})/2)$. This was carried out for each individual bucket and field plot.

The field plot experiment yielded paired data—six pairs (*X*_*i*_, *Y*_*i*_), *i* = 1 ... 6, in which *X*_*i*_ are cumulated emissions from plots treated with NNRB, and *Y*_*i*_ are cumulated emissions for control plots. This gives six ratios *R*_*i*_ = *X*_*i*_/*Y*_*i*_. Confidence intervals for the mean of the ratios, 1/6 Σ*R*_*i*_, for two time periods were made with a Student’s *t* distribution (assuming that the ratios were normally distributed). These confidence intervals were similar to confidence intervals found by the Fieller method for ratios of paired data and also by simple nonparametric bootstrapping^68^.

As the field bucket experiments did not yield paired data, flux reduction statistics are calculated as ratios of means, rather than means of ratios, of cumulated fluxes. Confidence intervals of these ratios were made by the Fieller method for unpaired data^69^ and by simple nonparametric bootstrapping (the results were similar). The 95% coverage of the Fieller confidence intervals was tested by numerical simulations and a bootstrap-calibration of the confidence level was made, with negligible effects on the confidence intervals.

The plots in Figs. 2, 4 and 5 were prepared using the packages Tidyverse (v2.0.0)^70^, Pracma (v2.4.2)^71^, ggbreak (v0.1.2)^72^, patchwork (v1.1.3)^73^ and scico (v1.5.0)^74^, in the R Studio software (v4.3.2)^75^. Colours used in the figures are, in general, from the scientific colour maps as described in ref. ^76^. The Fieller and bootstrap confidence intervals were calculated using Python (v3.11.5)^77^ with Scipy (v1.11.2)^78^ and Pandas (v2.1.1)^79^, and Julia (v1.9.3)^80^.

### Tracing CB-01 in digestate and soil

To quantify CB-01 cells in digestate and soil, we used qPCR with primers specific to members of the genus *Cloacibacterium* developed previously^81^. The primers 5′-TATTGTTTCTTCGGAAATGA-3′ (Cloac-001f) and 5′-ATGGCAGTTCTATCGTTAAGC-3′ (Cloac-001r) target a region of the 16S rRNA gene.

DNA was extracted with the DNeasy PowerSoil Pro Kit (Qiagen) according to the manufacturer’s protocol, except for the first step: bead beating of the cells was carried out at 4.5 m s^−1^ for 45 s in a FastPrep-24 (MP Biomedicals), instead of a vortex. To measure the concentration of DNA in the extract, we used a broad-range or high-sensitivity Qubit dsDNA Assay Kit (Thermo Fisher Scientific), depending on the expected concentration. The number of CB-01 16S rRNA gene copies in extracted DNA was quantified using a CFX96 Touch Real-Time PCR Detection System (Bio-Rad), running for 15 min at 95 °C followed by 40 cycles of denaturation (30 s at 95 °C), annealing (30 s at 55 °C) and elongation (45 s at 72 °C). The final concentration of the master mix contained 0.2 µM of each primer (Cloac-001f and Cloac-001r), and 1× HOT FIREPol EvaGreen qPCR Supermix (Solis BioDyne).

For calibration, we used DNA-extracted suspensions of washed cells containing 10^3^, 10^4^, 10^5^, 10^6^, 10^7^ and 10^8^ cells per millilitre, resulting in 2.4 × 10^1^–2.4 × 10^6^ 16S templates per PCR tube (taking dilution into account, and the fact that each genome of CB-01 contains three 16S rRNA genes). Results from the qPCR were analysed using the CFX Maestro 1.1 software (v4.1.2433.1219 from Bio-Rad). To enable the use of the Cq values to estimate copy numbers, we used the generalized reduced gradient solver in Excel to fit the model (equation (1)) to the data:

1 $$N=\,\frac{{N}_{T}}{{(2\times e)}^{{\rm{Cq}}}}$$

in which *N* is the initial number of 16S rRNA gene templates in the PCR tube, *N*_*T*_ is the number of amplicons per tube needed for signal detection (above background), *e* is the efficiency of the PCR amplification and Cq is the number of cycles needed for detection of a signal. The fitted parameters were *N*_*T*_ = 7.68 × 10^10^ copies per tube and *e* = 0.85 (85% efficiency).

An independent dataset was provided by running qPCR with the same primers on extracted DNA from suspensions of unwashed CB-01 cells (in nutrient broth) with densities 10^4^, 10^5^, 10^6^, 10^7^ and 10^8^ cells per millilitre. The log_10_ values of cell densities estimated by the Cq values were on average 104% of the expected value, with a standard deviation of 6%.

When using qPCR to estimate the CB-01 abundance in soil and digestate, inhibition of the polymerase can result in too high Cq numbers, hence resulting in underestimation of the gene abundance^82^. To investigate this, we spiked the different soils and the digestate with 10^9^ CB-01 cells per gram of soil dry weight and per millilitre of digestate, respectively, extracted DNA from 0.2 g soil and 0.2 ml digestate, and eluted to a 50-µl DNA solution for each material, which was then diluted in tenfold steps from 0 (undiluted) down to 1/10^7^. The results show a reasonable fit between model (predicted) and measured Cq values for all materials if diluting the extracted DNA to ≤1/10, except for the intermediate-pH clay loam (pH(CaCl_2_) = 6.13), which required dilution to ≤1/100 to eliminate inhibition (for further details, see Supplementary Fig. 2).

The result was used to approximate the lower limit for detection of CB-01 in soils and digestate: a cautious upper limit for Cq values to be trusted is 40 (that is, 34 templates per PCR tube; equation (1)). The polymerases were evidently inhibited by using undiluted DNA in the reaction (Supplementary Fig. 2); hence, a 1/10 dilution of the extracted DNA is needed for all soils except soil I, for which 1/100 dilution is required. This means that the PCR tube can maximally be loaded with DNA from 0.8 mg soil (0.08 mg for soil I) and 0.8 µl digestate. This implies a limit of detection around 4.3 × 10^4^ templates per gram of soil (4.3 × 10^5^ for soil I owing to dilution to 1/100) and per millilitre of digestate, or 1.4 × 10^4^ CB-01 genomes per gram of soil and per millilitre of digestate (as the genome contains three copies of the 16S rRNA gene).

The real limit of detection for a CB-01 inoculum in soil and digestate could be higher than this, if indigenous genes are amplified with the primers. This was tested by running PCR on soil and digestates that had not been spiked with CB-01, along with analysing spiked samples in various experiments. The results are summarized in Supplementary Fig. 2. As there were several tubes with a negative result (Cq > 40), average values cannot be calculated. A cautious judgement would be that the ‘background’ PCR signal of the soil is Cq = 39–38, which is equivalent to 67–107 templates per PCR tube, or 21–36 CB-01 genomes per tube. For all soils except I, we used the Cq values for the PCR tubes loaded with 1/10 dilutions, which were thus loaded with DNA from 0.8 mg soil. For these, the background PCR signal is equivalent to 2.6–4.8 × 10^4^ CB-01 genomes per gram, and 10 times higher for soil I (owing to 1/100 dilution of the DNA from this soil). For digestate, the average Cq was 31.98 (Fig. 2), which means that the untreated digestate contains 3.2 × 10^6^ CB-01 16S templates per millilitre, or 1.1 × 10^6^ CB-01 genomes per millilitre.

### Survival of CB-01 in soil

#### Laboratory experiment

A soil incubation experiment was designed to assess the survival of CB-01 in soil, vectored by digestate, under constant temperature and moisture conditions, and without any subsequent incorporation of digestate (thus contrasting with the field bucket experiment, Fig. 2). CB-01 was first grown to about 6 × 10^9^ cells per millilitre in autoclaved, aerated and pH-adjusted digestate (as for the field experiments). Neutral-pH clay loam soil (soil N, see Extended Data Fig. 6) was portioned into a set of 50-ml Falcon tubes (9.4 g soil dry weight, moisture content = 0.5 ml g^−1^ soil dry weight). To each tube, 4.2 ml sterile water and 0.85 ml digestate (with CB-01) were dripped onto the soil. The tubes were stored in a dark moist chamber at 15 °C, with loose lids to allow exchange of air. Control tubes received only sterile water. At intervals, two replicate tubes were frozen (−20 °C) for quantification of CB-01 16S rRNA gene abundance by qPCR as described above.

#### Field plot experiment

From each individual plot (Fig. 5) we took three replicate soil samples, 9 and 280 days after fertilization, for quantification of CB-01 abundance by qPCR.

### Extrapolating to national emission reductions

We use the emissions quantified with the GAINS model^48,49^ for 2030 in Europe to estimate the possible reductions of the measure.

The experiments described in this paper demonstrate marked emission reductions on all soils tested, over extended periods. The strongest reductions have been seen for the initial N_2_O peak immediately after fertilization, but NNRB has shown to remain active over a period of 90 days. Cumulated emissions over the whole period have been reduced by at least 41% (for clay loam soils), up to 95% reduction. We may disregard the case of the smallest reduction as the emissions from these soils are also rather small, but the organic loam soils (55% reductions) need to be considered. Consistent with the uniform emission factor used in GAINS (from IPCC^50^) of 1% of N applied to be emitted as N_2_O for all conditions of crops, soil or type of fertilizer added, a uniform reduction factor of 60% of emission reductions due to NNRB, which we consider a conservative estimate, was also applied. In Extended Data Table 3, emission reductions are shown by European country for 2030 if emissions from application of liquid manure alone are reduced by 60%. This assumption is based on the understanding that liquid manure can easily be treated in biodigesters. The authors of ref. ^83^ assume, for the purpose of methane abatement, that anaerobic digestion becomes profitable only for large agricultural entities of at least 100 livestock units. According to GAINS numbers, this concerns 70% of all farms in Europe, which more probably reflect liquid rather than solid manure systems, so the above estimate remains valid for the main fraction of liquid manure available. Indirect emissions as well as other soil emissions due to grazing, mineral fertilizer additions or application of farmyard manure (solid manure systems) have been left unchanged. Note that the GAINS model (in agreement with IPCC^50^) does not account for potentially increased emissions due to dry periods or freeze–thaw cycles (the latter considered to potentially contribute as much as 17–28% to global soil emissions^84^) but it covers increased emissions from cropping histosols.

Under these assumptions, total N_2_O emissions from Europe decrease by 2.7% owing to NNRB introduced. This figure is higher in countries that have a high share of liquid manure systems in their agriculture; hence, for EU27 (27 EU member countries) the corresponding figure is 4.0%, if NNRB were used for all manure nitrogen applied from liquid manure systems.

If it were possible to extend the NNRB technology, using solid manure and plant residues as substrates and vectors, we speculate emission reductions could be achieved for all mineral and natural fertilizer actively applied on fields. Ongoing work has shown that although *Cloacibacterium* sp. CB-01 grows to high cell densities in plant residues, new strains that grow in manure have been enriched and isolated (K. R. Jonassen and S. H. W. Vick, unpublished results). Although further development will be needed to implement this, it is relevant to estimate their impacts. Applying NNRB also to these other substrates at the same reduction efficiency could decrease European emissions as well as EU27 emissions by about a quarter (24% and 23%, respectively). For agricultural emissions alone, this means that roughly a third (31%) could be eliminated. For this calculation, we assume that indirect emissions from agriculture (due to re-deposition of ammonia released from fertilizers, or due to nitrate leaching), manure-management-related emissions and emissions from histosols remain unaffected.

It needs to be pointed out that an emission reduction of 60% as derived here for NNRB is much larger than emission reductions typically reported for N_2_O abatement measures. For example, GAINS assumes nitrification inhibitors to be able to reduce emissions by as much as 38%, and high-tech mechanical fertilizer-saving technologies (‘variable rate application’) to be able to save only 24% of the emissions^48^. Of note, the percentage reduction of N_2_O emission by the NNRB technology is plausibly unaffected by ‘variable rate application’ and nitrification inhibitor, as the target for NNRB is to reduce the N_2_O/N_2_ product ratio of denitrification, whereas the two others target the concentration of NO_3_^−^ and nitrification, respectively.

### Effect of CB-01 on the soil microbiome

Microbial community composition was examined by amplicon sequencing of the 16S rRNA gene V3–V4 region. Purified DNA from soil samples was sent to Novogene Europe for amplification, library preparation and sequencing to generate 250-base-pair paired-end reads using the Illumina Novoseq platform. Reads, after primer removal, were processed using GHAP (v2.4)^85^, an in-house amplicon clustering and classification pipeline built around Usearch (v11.0.66)^86^, the RDP classifier (v2.13)^87^ and locally written tools for generating operational taxonomic units (OTU) tables. Reads were processed using default quality control and trimming parameters. Clustering was carried out at both 97% and 100% similarity to generate OTUs and zero-radius OTUs (zOTUs), respectively. The 16S rRNA gene sequence of *Cloacibacterium* sp. CB-01 (GCA_907163125) was then matched against the OTU and zOTU representative sequences using the Usearch usearch_global command at 97% similarity and 99% similarity, respectively, to determine which OTU and zOTUs circumscribe the *Cloacibacterium* sp. CB-01 inoculant. From visual inspection it appeared that two zOTUs (zotu45 and zotu611) may circumscribe *Cloacibacterium* sp. CB-01 owing to shared abundance profiles and taxonomic classifications. To confirm that these two zOTUs both matched to *Cloacibacterium* sp. CB-01, the two representative sequences were BLAST-searched^88^ against the *Cloacibacterium* sp. CB-01 genome, and it was observed that both zOTU sequences matched closely to two separate regions of the genome, presumably harbouring multiple slightly divergent copies of the 16S rRNA gene. To confirm this, the two 16S rRNA genes from the *Cloacibacterium* sp. CB-01 genome were matched back against the zOTU representative sequences using the usearch_global command at 99% similarity, at which they matched to both zotu45 and zotu611, separately. Owing to this, zotu45 and zotu611 were combined for downstream analyses.

To assess the impact of the various treatments on the soil microbial communities, α- and β-diversity measures were calculated for microbial communities from all samples using the OTU tables generated above. OTU tables were first modified by removing the OTU circumscribing *Cloacibacterium* sp. CB-01 (OTU_27) before rarifying the tables to 72,846 reads per sample using the Usearch otutab_rare command. Shannon’s^89^ and Simpson’s^90^ diversity indices were calculated using the Usearch -alpha_div command and β-diversity measures were calculated using the Usearch -beta_div command. Jaccard’s dissimilarity measures^91^ were then used to generate multidimensional scaling plots using the Scikitlearn MDS module^92^.

The β-diversity as shown by Jaccard’s dissimilarity measures indicated that early during the soil incubation period there is greater between-sample variation both within treatments and between soils treated with live CB-01 and those treated with water or dead CB-01, indicating an effect of CB-01 on the soil microbial communities (Extended Data Fig. 7a). This effect, however, disappears by the final time point, at which samples from live-CB-01-, dead-CB-01- and water-treated soils cluster together, suggesting that the effect of live CB-01 on native soil microbial communities is transient and microbial soil communities are not affected in the longer term by the addition of live CB-01. It should be noted that the effect over time throughout the experiment is also a much larger source of microbial community variation than the addition of live CB-01 cells, presumably owing to disturbances to the soil from digging, sieving and packing of pots. Similarly, no systematic effects are observed on the α-diversity of soil microbial communities throughout the experiment indicating that the CB-01 treatment does not reduce the complexity or evenness of soil microbial communities when added to soils with digestate organic matter as can be seen in the Shannon and Simpson diversity measures of samples taken throughout the experiment (Extended Data Fig. 7b,c).

### Search for antibiotics resistance genes and pathogenicity in CB-01

Microorganisms produce secondary metabolites crucial for diverse microorganism–microorganism interactions, enhancing survivability and competitive fitness through antagonistic effects on competitors under limited growth conditions. This array of metabolites, including antibiotics, toxins, pigments, growth hormones and anti-tumour agents, can also contribute to virulence and human pathogenicity. Such traits, if encoded in the inoculant’s genome, would restrict the use of such organisms as inoculants in agricultural soil. Likewise, the use of an inoculant would be restricted if its genome contains antibiotic resistance genes.

We checked CB-01 for such traits, scrutinizing its assembled draft genome^7^ in Pathogenfinder (v1.1)^93^ and ResFinderFG (v2.0)^94^, using standard settings. This revealed no evidence of human pathogenicity or antimicrobial resistance genes.

### Reporting summary

Further information on research design is available in the Nature Portfolio Reporting Summary linked to this article.

## Online content

Any methods, additional references, Nature Portfolio reporting summaries, source data, extended data, supplementary information, acknowledgements, peer review information; details of author contributions and competing interests; and statements of data and code availability are available at 10.1038/s41586-024-07464-3.

### Supplementary information

Supplementary Information

Reporting Summary

Peer Review File

## Figures and Tables

**Extended Data Table 1:**
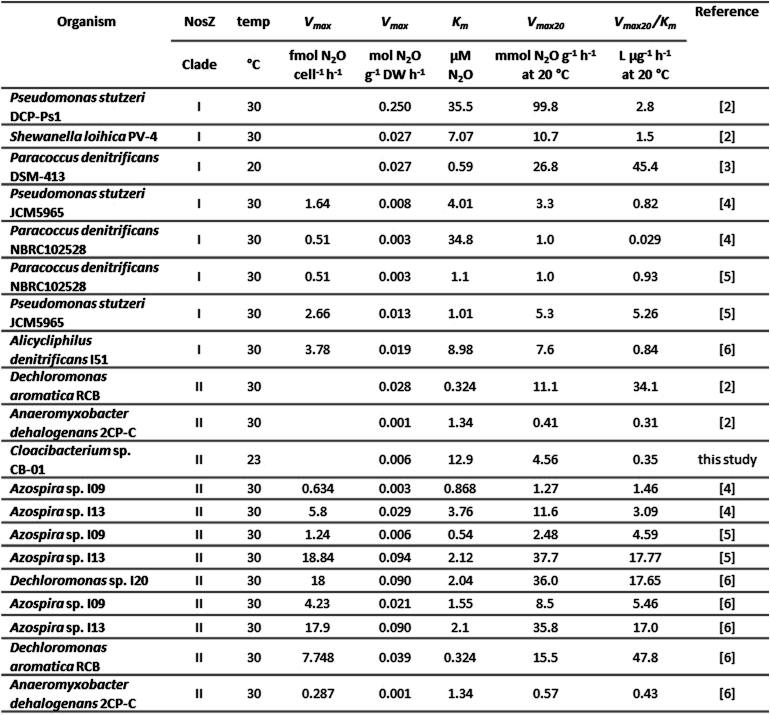
Comparison of biokinetic parameters for CB-01 and other N_2_O-respiring bacteria

The maximum rates of N_2_O-respiration (*V*_*max*_), and the affinity for N_2_O, normally expressed as the half saturation concentration (*K*_*m*_), have been measured in various N_2_O-respiring strains to assess and compare their capacity to scavenge N_2_O in soil. A plausible way to rank strains according to their capacity to reduce N_2_O at low N_2_O-concentrations is to calculate their *V*_*max*_/*K*_*m*_ ratios, since this approximates the slope of the rate against N_2_O-concentration ([N_2_O]) at very low concentrations ([N_2_O] « *K*_*m*_). In the table below, we have listed *V*_*max*_ and *K*_*m*_ determined for a range of N_2_O-respiring strains. Some report *V*_*max*_ as fmol N_2_O cell^−1^ h^−1^, and others report it as mol g^−1^ cell dry weight h^−1^. To enable a comparison, we have converted all *V*_*max*_ values to mol N_2_O g^−1^ cell dry weight h^−1^, assuming a cell volume = ~0.6 µm^3^ and 200 fg dry weight per cell^97^, and converted all values to V_max_ at 20 °C assuming the rates increase exponentially with temperature by a factor of 2.5 per 10 °C increase in temperature (Q_10_ = 2.5). The calculated *V*_*max20*_*/K*_*m*_ values are plotted against *V*_*maxN20*_ in Fig. 1b in the main paper. refs. ^38,60,97–100^.

**Extended Data Table 2:**
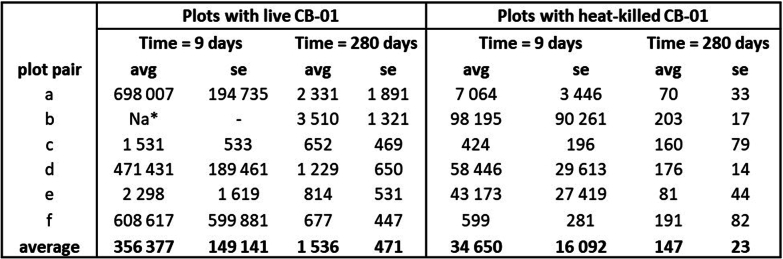
Estimated abundance of CB-01 genomes in the field plot experiment

The numbers are the average genome abundance based on qPCR analysis of 3 replicate samples for each indivdual plot (unit: 10^3^ genomes g^−1^ soil dry-weight), taken 9 and 280 days after fertilization with CB-01 containing digestate (live or heat killed). Initial numbers of CB-01 (at time 0) was ~6 × 10^8^ cells g^−1^ soil dry-weight, as calculated from the measured O_2_-consumption during cultivation in the digestate (Extended Data Fig. 5a–c), corrected for the low growth yield in digestate (Extended Data Fig. 1b), and the amount of digestate per g soil dry-weight). The genome abundance in the soil samples taken 9 days after fertilization are remarkably variable, suggesting a fast initial decline in two of the plots with live CB-01 (plot c and e), at rates comparable to the rapid initial decline in the laboratory incubation experiment (Fig. 6 in the main paper), tentatively ascribed to protozoal grazing. The variability suggests a patchy distribution of protozoa in the field. Of note, the genome abundance after 280 days in the plots with live CB-01 are in some agreement with the first order decline rates estimated for the field bucket experiment: The decline in genome abundance from 3.56 × 10^8^ at time = 9 days to 1.5 × 10^6^ at time 280 days implies an average first order decay rate of 0.02 d^−1^ ( = average half life of 34 days). *samples lost

**Extended Data Table 3:**
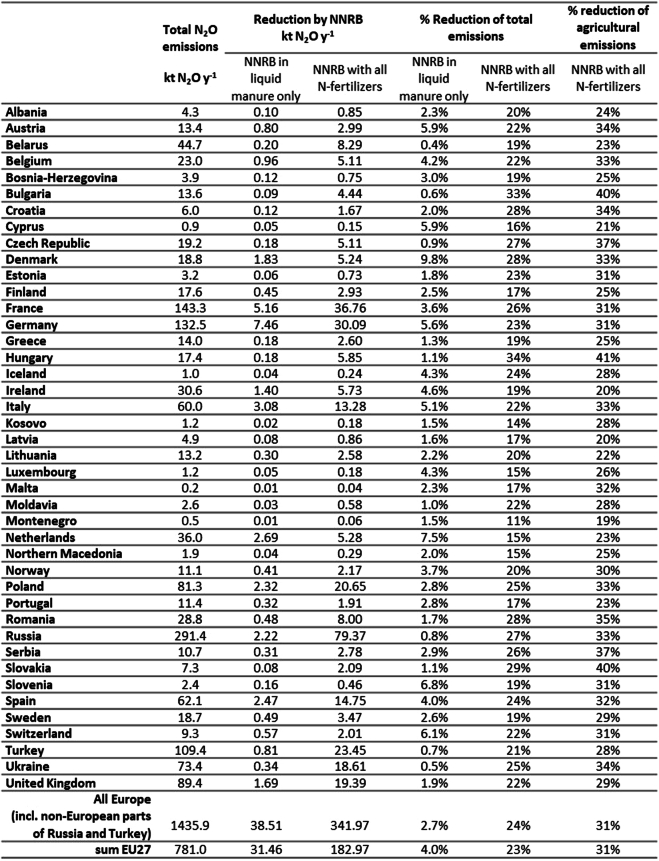
Potential emission reductions as a consequence of implementing NNRB measures

(anthropogenic emissions in kt N_2_O per year projected for 2030).

## Data Availability

Data that support the findings reported in this study are available at Figshare (10.6084/m9.figshare.25130507)^95^. The assembled draft genome of CB-01 was downloaded from the European Nucleotide Archive (accession number GCA_907163125). 16S rRNA sequence data were deposited in the National Center for Biotechnology Information Sequence Read Archive database under accession number PRJNA878624.

## Code availability

Code for calculating confidence intervals of ratios of time-integrated fluxes are available at https://github.com/larsmolstad/cloacipaper_stats.
