# Supplementary material for: Unlocking bacterial potential to reduce farmland N2O emissions
Source: Nature. 2024 May 29;630(8016):421–8. doi: 10.1038/s41586-024-07464-3 (PMC11168931; doi:10.1038/s41586-024-07464-3)
Supplement: Supplementary file 1 — Supplementary Information [file 41586_2024_7464_MOESM1_ESM.pdf]

nature portfolio

https://doi.org/10.1038/s41586-024-07464-3

Supplementary information

Unlocking bacterial potential to reduce farmland N_2O emissions

In the format provided by the
authors and unedited

Supplementary Information, Hiis et al: Unlocking potential to reduce farmland N_2O emission.

Fig. 1 shows the methods and equipment used in field experiments.
Fig. 2 shows the validation of qPCR as a method to detect CB-01 cells in different soils.

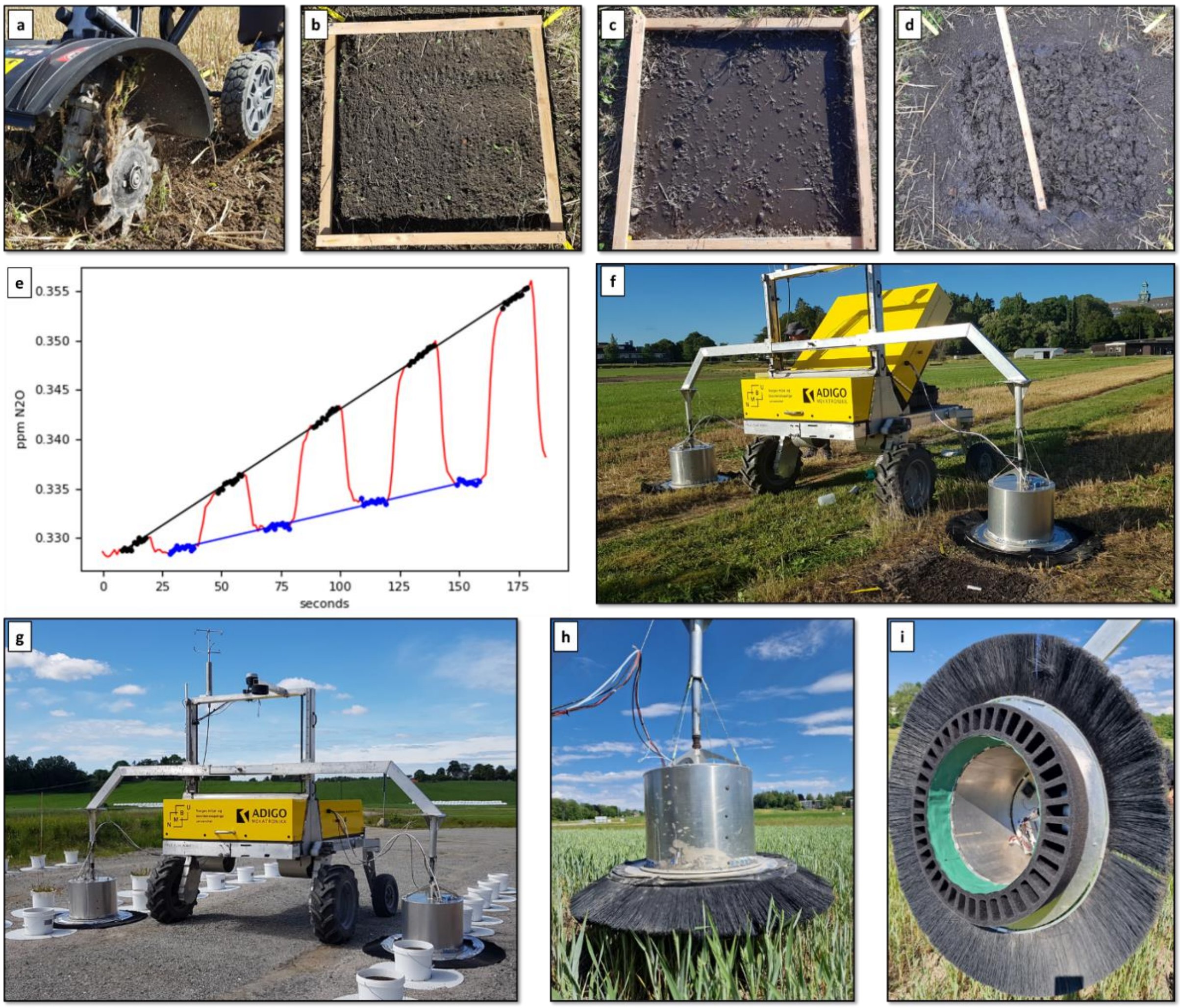

Fig. 1 The Field experiment. <u>**Panels a-d**</u> show field plot preparations: The top ~10 cm of the soil was first loosened by a cultivator (panel a). After evening out the surface with a rake, we placed a 0.7 X 0.7 m frame onto the surface (panel b), and poured digestate onto the surface within the frame (panel c). One day later the top soil (0-10 cm) was harrowed by a hand held tool (panel d). <u>**Panels e-i**</u> show details of the Field Flux Robot (FFR). The FFR operates two chambers (diameter 50 cm, height 50 cm), which are lowered onto the field plots (panel f) or over the buckets (panel g). The chambers are equipped with cellular foam with gas tight flexible rubber coating which is compressed by deployment, and a circular brush skirting to function as a wind break (panels h-i). The concentrations of N_2O and CO_2 in the two chambers are measured by circulating the chamber-air (intermittently for each chamber) via a Tunable Diode Laser N_2O/CO-analyzer (DLT-100, Los Gatos Research, CA, USA) and a CO_2/H_2O infrared gas analyzer (LI-840A, LI-COR Biosciences, NE, USA), throughout a deployment time of 3 minutes. <u>**Panel e**</u> shows the measurement during a 3 minutes deployment. The jagged shape of the curve is due to valves in the robot switching every 20 seconds, alternating between circulating air from the left and the right chamber through the laser instrument. The two straight lines are regression lines, the slopes of which are used to calculate the emission fluxes. (In this example, blue dots: soil with live CB-01, black dots: soil with dead CB-01.)

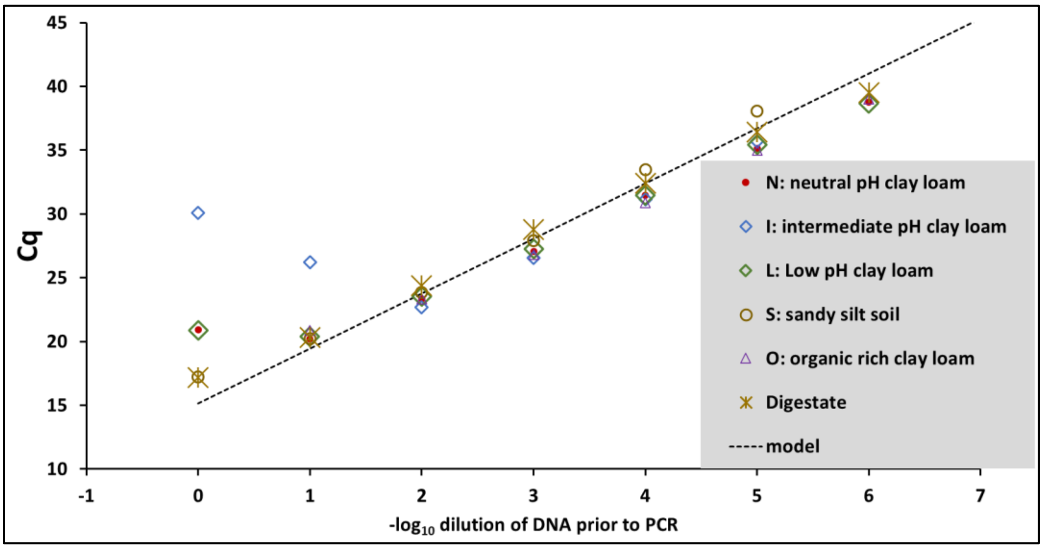

Material	Total number of samples	Number of samples with Cq<40	Average Cq for samples with Cq<40
**N:** Neutral-pH clay loam	8	2	39.33
**I:** Intermediate-pH clay loam	12	7	39.48
**L:** Low-pH clay loam	3	1	37.78
**S:** Sandy silt soil	3	0	-
**O:** Organic-rich clay loam	3	2	39.8
Digestate	3	3	31.98

Fig. 2 Inhibition of qPCR, and assessment of background in soil and digestate.
Top panel: Soils and digestate were spiked with CB-01 cells (10^9 cells g^{-1} soil dw and mL^{-1} digestate, respectively). DNA was extracted from 0.2 g soil and 0.2 mL digestate, eluted in a 50 µL volume. From undiluted, and 10-fold dilutions of this, 2 µL were transferred to PCR tubes for amplification of CB-01 16S rRNA genes. The plot shows the Cq values plotted against dilution, together with the model $N = N_T/(2\ e)^{Cq}$ as parameterized (see text): $\boldsymbol{N_T}$ = 7.68 × 10^{10} copies per tube and *e* = 0.85.
The table shows the result of the inspection of the "background" in digestate and soils without CB-01 added. The table shows the total number of samples analyzed for each soil and for the digestate, the number of samples with Cq > 40 (estimate not available), and the average for those < 40. The results suggest that the background Cq values are > 38, which implies that the background is < 1.25 × 10^5 16S-templates g^{-1}, hence < 4.2 × 10^4 CB-01 genomes g^{-1} soil. The background value of digestate is a bit higher, with a Cq of 31.98, corresponding to about 3.2 × 10^6 16S rRNA gene templates mL^{-1}, or 1.1 × 10^6 CB-01 genomes mL^{-1} digestate.
